# Stereochemistry of Chiral 2-Substituted Chromanes: Twist of the Dihydropyran Ring and Specific Optical Rotation

**DOI:** 10.3390/molecules28010439

**Published:** 2023-01-03

**Authors:** Bei-Bei Yang, Fan Gao, Ya-Dong Yang, Ru Wang, Xin Li, Li Li

**Affiliations:** Beijing Key Laboratory of Active Substances Discovery and Druggability Evaluation, Institute of Materia Medica, Chinese Academy of Medical Sciences & Peking Union Medical College, Beijing 100050, China

**Keywords:** 2-substituted chromanes, stereochemistry, specific optical rotation, density functional theory, helicity

## Abstract

Chiral 2-substituted chromanes are important substructures in organic synthesis and appear in numerous natural products. Herein, the correlation between specific optical rotations (SORs) and the stereochemistry at C2 of chiral 2-substituted chromanes was investigated through data mining, quantum-chemical calculations using density functional theory (DFT), and mechanistic analyses. For 2-aliphatic (including acyloxy and alkenyl) chromanes, the *P*-helicity of the dihydropyran ring usually corresponds to a positive SOR; however, 2-aryl chromanes with *P*-helicity tend to exhibit negative SORs. 2-Carboxyl (including alkoxycarbonyl and carbonyl) chromanes often display small experimental SORs, and theoretical calculations for them are prone to error because of the fluctuating conformational distribution with computational parameters. Several typical compounds were discussed, including detailed descriptions of the asymmetric synthesis, absolute configuration (AC) assignment methods, and systematic conformational analysis. We hope this work will enrich the knowledge of the stereochemistry of chiral 2-substituted chromanes.

## 1. Introduction

**Figure 1:**
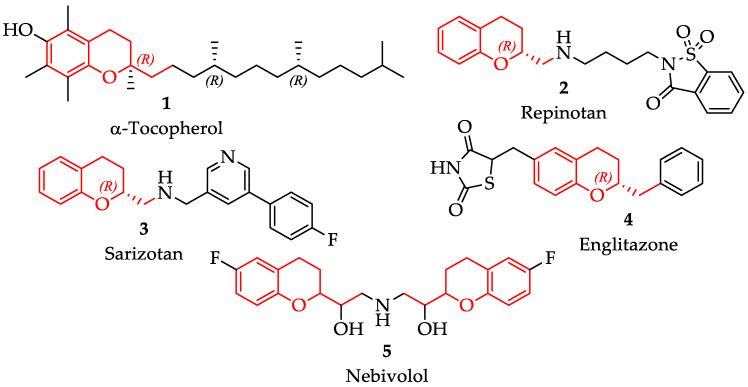
Representative bioactive 2-substituted chromanes.

Chiral 2-substituted chromanes (dihydrobenzopyrans) are biologically active compounds that are ubiquitous in a variety of natural products [1,2]. They display a broad spectrum of biological activities, such as the fat-soluble vitamin α-tocopherol (**1**, Figure 1) [3], the potent 5-HT1A receptor agonists repinotan (**2**, Figure 1) [4] and sarizotan (**3**, Figure 1) [5], the antidiabetic agent englitazone (**4**, Figure 1) [6], and the β1-selective adrenergic receptor blocker nebivolol (**5**, Figure 1), which is used as an antihypertensive drug [7]. Although nebivolol is used clinically as a racemic mixture, the dextro-isomer exhibits β-adrenoceptor blocking activity over a thousand times greater than that of the levo-isomer [8].

**Scheme 1:**
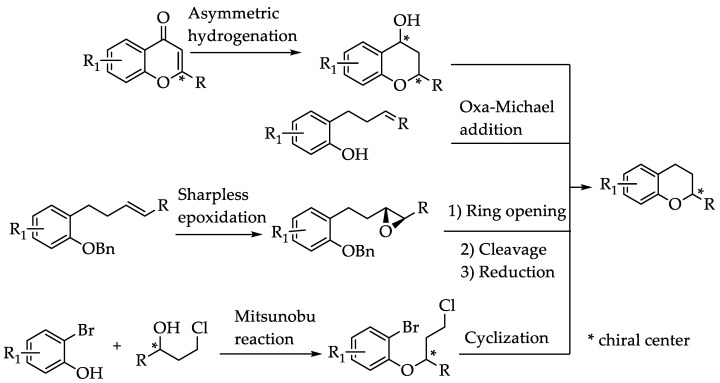
Representative approaches to prepare optically active 2-substituted chromanes.

The development of facile and efficient asymmetric synthetic approaches for chiral chromanes has been extensively investigated, and various strategies are currently available (Scheme 1). Asymmetric hydrogenation of chromones has been established to afford chiral 2-substituted chromane moieties with low economic costs and high efficiency [9]. Phenol substrates bearing (E)-α,β-unsaturated ketone moieties were shown to produce the chromane skeleton via intramolecular oxy-Michael addition [10]. Dinda and coworkers established a facile enantioselective synthetic route to afford chiral chromane derivatives in excellent yields and with suitable enantioselectivity by employing phenolate ion-mediated intramolecular epoxide ring-opening reactions [11]. The C2 stereocenter is also accessible via a Mitsunobu inversion reaction with 2-bromophenol and an appropriately substituted chiral halopropanol, followed by cyclization to form the dihydrobenzopyran ring [12]. In addition, the kinetic resolution of racemic chromanes is another common method of obtaining the desired optical isomers [13].

Although considerable effort has been expended to develop versatile strategies for the asymmetric synthesis of chiral 2-substituted chromanes, much less effort has been devoted to studying the stereochemistry of the prepared compounds. Their absolute configurations (ACs) were either assigned by performing an X-ray analysis of derivatives [14] or by comparing the specific optical rotation (SOR) with that of known compounds [15] or were only tentatively assigned [16,17].

Recently, chiroptical spectroscopic methods, including electronic circular dichroism (ECD) [18,19,20], vibrational circular dichroism (VCD) [21,22], and SOR [23,24,25], have been widely applied to determine the ACs of chiral drugs or natural products. For chiral chromanes, a helicity rule for the interpretation of ECD spectra was proposed by Snatzke et al., and the scope of its application was discussed in depth [26,27,28]. Nevertheless, since SOR at 589.3 nm ([α]_D_) is indispensable for the characterization of chiral molecules, the collection of available SOR data for chiral 2-substituted chromanes exceeds that of ECD data. Moreover, some conflicting SOR data were reported for a compound with a definite configuration [9,12,29]. This fact inspired us to investigate the correlation between the SOR and AC of chiral 2-substituted chromanes. Furthermore, chiral centers other than the C2 atom might exert significant effects on the SORs, and our study was mainly focused on compounds with only C2 chiral center.

## 2. Results

### 2.1. Data Mining and Analysis

**Figure 2:**
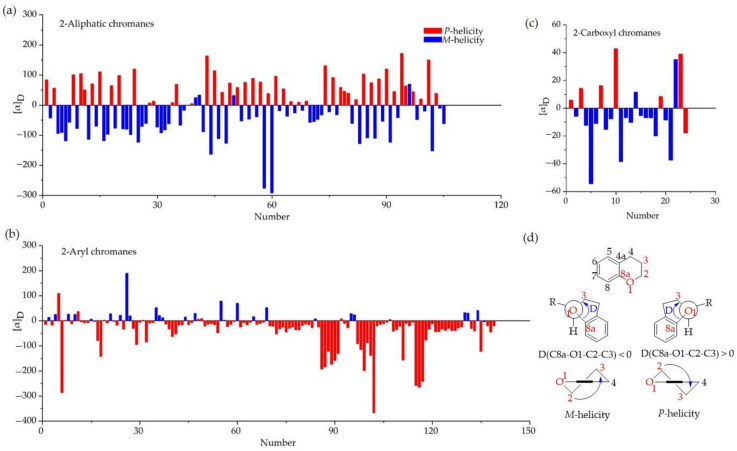
Histograms of the SORs of various chiral 2-substituted chromanes: (**a**) 2-aliphatic chromanes, (**b**) 2-aryl chromanes, and (**c**) 2-carboxyl chromanes; red: [α]_D_ of chromanes in *M*-helicity, blue: [α]_D_ of chromanes with *P*-helicity. (**d**) Graphical representation of the correlation between the *P*-/*M*-helicity of the dihydropyran ring and the torsion angle D(C8a-O1-C2-C3).

First, chiral 2-substituted chromanes with definite, absolute configurations and their [α]_D_ values were collected from the literature, which are listed in Tables S1–S3. Most of the experimental data were measured in chloroform or methanol. Before analysis, it is necessary to assess the reliability of these raw data. According to the documented [α]_D_ and concentration, the α value was calculated, assuming a path length of 1 dm. Data with absolute α values less than 0.005 were abandoned, considering that the accuracy of optical rotation measurement ranged from ±0.001 to ±0.01. In total, 241 optically active 2-substituted chromanes, corresponding to 269 optical isomers with definite configurations and 316 SOR data points, were collected (Tables S1–S3). It is interesting to find that most 2-aryl or 2-carboxylic chromanes are levorotatory (Figure 2). The absolute configurations of these compounds were assigned explicitly in the original reports.

All these compounds were classified by the type of substituents at the C2 atom into the aliphatic group (including acyloxy and alkenyl, type I), the aryl group (type II), and the carboxyl group (including alkoxycarbonyl and carbonyl, type III). The C2 substituents were hypothesized to adopt an equatorial position of the half-chair conformation, and the twist of the dihydropyran ring corresponded exclusively to the C2 stereochemistry on this premise. Thus, the chromane helicity was characterized by the torsion angle D(C8a-O1-C2-C3), with positive values indicating *P*-helicity and negative values indicating *M*-helicity. As for typical helical molecules [30], a clear correlation between the sign of the SORs and chromane helicity was observed except for a few outliers, which are discussed below.

For 2-aliphatic (type I) or 2-carboxyl (type III) chromanes, the *P*-helicity of the dihydropyran ring generally corresponds to a positive SOR. The SOR values of chromanes in type I mainly ranged from 50 to 150, with several points located in the range of 200–300. The 2-carboxyl chromanes exhibited smaller SOR values, and the available data were relatively small compared with the other two groups. The number of SOR values for the 2-aryl chromanes was the largest, and most absolute values were less than 100. In contrast to type I and III compounds, 2-aryl chromanes with *P*-helicity generally tend to have negative SORs, as determined from the analysis of 139 molecules corresponding to 163 SOR data points.

Regarding the sign inversion of SORs observed for 2-aryl chromanes, the exact underlying mechanism is not clear, and the possible causes might be the high polarizability [31,32] and chromophore property of the aryl group, which might lead to electronic transition coupling with the chromane core. In addition, the nature and position of substituents on the phenyl ring were also influential in some cases.

The premise of this correlation is that the C2 substituent is located along the equatorial bond of the half-chair conformation, as shown by the coupling constants between H2 and two H3 atoms in the ^1^H-NMR spectra (Table S3). For those 2,2-disubstituted chromanes, the smaller methyl group is often located in the equatorial position [28], resulting in an inverse relationship between SOR and C2 stereochemistry.

Some 2-substituted chromanes showed minor SOR values due to both structural factors and nonstructural factors. The structural factors of the molecules led to the existence of conformations of opposite optical rotation signs, resulting in a small inherent SOR. Regarding nonstructural factors, the possible reasons might be the low enantiomeric excess (ee) of the tested samples, measurement error, simple trivial error, or incorrect previous AC assignments.

### 2.2. Verification of the Correlation

#### 2.2.1. Correlation between SOR and C2 Stereochemistry

Both quantum-chemical calculations using density functional theory (DFT) and mechanistic analyses were conducted to verify the association between the helicity of the dihydropyran ring and the sign of the SORs of chiral 2-substituted chromanes. Since different computational approaches might yield misleading results [33], several combinations of computational parameters, B3LYP [34]/Aug-cc-pVDZ [34]//B3LYP/6-311G(d,p) (the BL approach) and M06-2X [35]/Aug-cc-pVDZ//M06-2X/TZVP (the M6 approach), together with the polarized continuum model (PCM) [36] or solvation model based on density (SMD) [37] were tested to compare their uniformity.

**Figure 3:**
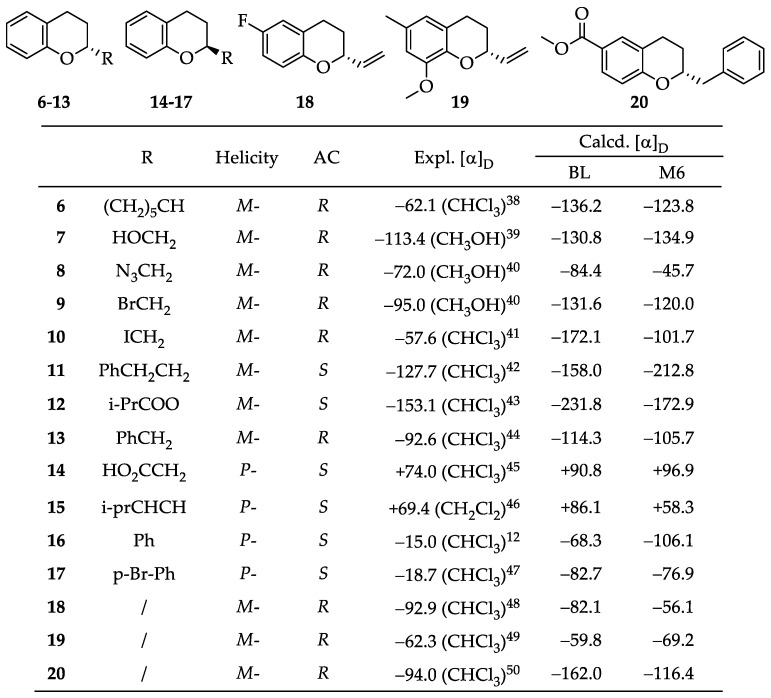
Experimental and calculated SORs for typical 2-substituted chromanes. BL: PCM/B3LYP/Aug-cc-pVDZ//B3LYP/6-311G(d,p), M6: PCM/M06-2X/Aug-cc-pVDZ//M06-2X/TZVP. Experimental SOR data were collected from references [12,38,39,40,41,42,43,44,45,46,47,48,49,50].

Much to our delight, the calculated SORs using all the tested approaches possessed the same sign as that predicted from the correlation, albeit the values varied with the computational parameters (Figure 3).

Next, several chiral 2-substituted chromanes with or without substituents on the phenyl group were taken as typical examples to investigate the correlation and discuss the possible influencing factors.

#### 2.2.2. 2-Methylchromane

**Scheme 2:**
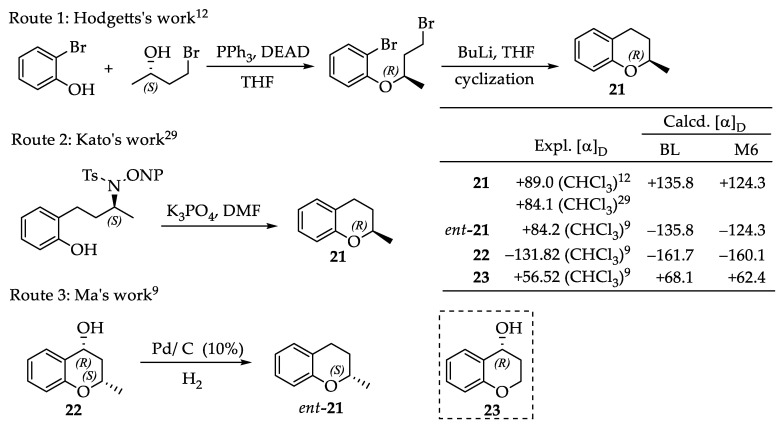
Synthetic routes for compounds **21** and *ent*-**21** [9,12,29].

2-Methylchromane (**21**, *ent*-**21**) is one of the simplest 2-substituted chromanes, and it is obtained through various approaches (Scheme 2).

In 2005, Hodgetts synthesized compound **21** using an intermolecular Mitsunobu reaction of homochiral halopropanol and 2-bromophenol followed by cyclization [12]. Five years later, compound **21** was prepared with an inversion of the configuration through a substitution reaction of an amine group by Kato and coworkers [29]. Both groups reported [α]_D_ values with the same positive sign and similar magnitude, namely, ${\left[\alpha \right]}_{D}^{22}$ (+89.0, c 1.0, CHCl_3_) and ${\left[\alpha \right]}_{D}^{25}$ (+84.1, c 0.98, CHCl_3_), respectively. Nevertheless, *ent*-**21** was recently reported to also display a positive ${\left[\alpha \right]}_{D}^{20}$ (+84.2, c 0.47, CHCl_3_) value [9].

The BL- and M6-calculated [α]_D_ values for compound **21** were +135.8 and +124.3, respectively. As expected, conformers with 2-methyl groups adopting equatorial positions prevailed overwhelmingly (>85%) in the conformational equilibrium mixture, regardless of the computational parameters. The [α]_D_ values of substrate **22** were also predicted theoretically, reproducing the experimental data very well. For compound **22**, the 4-hydroxy group did not change the correlation between the stereochemistry at C2 and the sign of SOR but increased the magnitude of the SOR value. Interestingly, (*R*)-4-hydroxyl chromane (**23**) displayed positive experimental and calculated SORs with values of +56.52 and +62.4 (M6) in CHCl_3_, respectively. Thus, the negative SOR of compound **22** was due to the existence of a 2*S*-methyl group, which determined the helicity of the chromane ring.

As shown in Figure 3, a correlation between the helicity of the dihydropyran ring and the sign of the SORs was observed for 2-cyclohexyl (**6**), 2-hydroxymethyl (**7**), 2-azidomethyl (**8**), 2-halomethyl (**9** and **10**), 2-phenylethyl (**11**), 2-isobutyryloxy (**12**), 2-carboxymethyl (**14**), and 2-benzyl (**20**) chromanes, indicating that these substituents do not influence the chromane helicity.

#### 2.2.3. 2-Vinylchromane

**Scheme 3:**
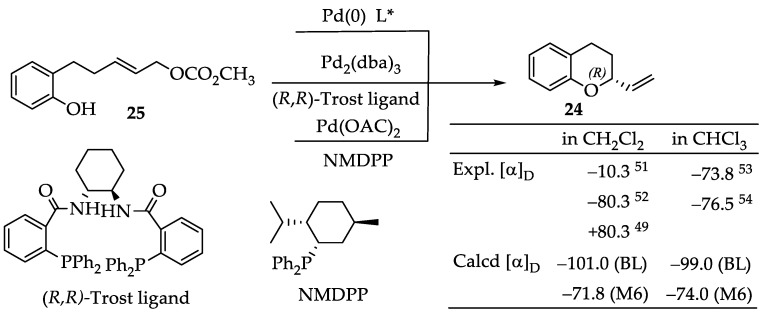
AAA reaction in the presence of different chiral ligands [49,51,52,53,54].

2-Vinylchromane (**24**) was obtained through asymmetric cyclization of allyl carbonate substrate **25** via asymmetric allylic alkylation (AAA) reactions (Scheme 3) by Labrosse and colleagues in 1999 [51].

Compound **24** displayed a negative [α]_D_ value (−10.3) in dichloromethane and was assigned the *R* configuration by conversion into putative (*R*)-2-hydroxymethyl chromane (**7**, Figure 3) [51]. The [α]_D_ value of the latter in methanol was reported to be −113.4, consistent with the correlation between SOR and C2 stereochemistry, and was further verified by DFT calculations in this work. In 2003, compound **24** with 84% ee was synthesized via a Pd-catalyzed AAA reaction in the presence of the (*R*,*R*)-Trost ligand, resulting in a [α]_D_ value of −80.3 in dichloromethane [52]. One year later, a chiral monophosphine ligand (NMDPP) was applied in the asymmetric synthesis of compound **24** via the AAA reaction, producing positive [α]_D_ data [49].

A vinyl group is larger than a hydrogen atom; thus, the *M*-helicity of chromane and a negative SOR were anticipated for compound **24**. Theoretical calculations yielded negative [α]_D_ values in dichloromethane of −101.0 and −71.8 using the BL and M6 approaches, respectively. The chloroform solution of compound **24** was recently reported to also exhibit negative [α]_D_ values (−73.8, c 1.0; −76.5, c 0.27) [53,54], indicating that the helicity of the dihydropyran ring was maintained in different solvents of similar structural types.

**Figure 4:**
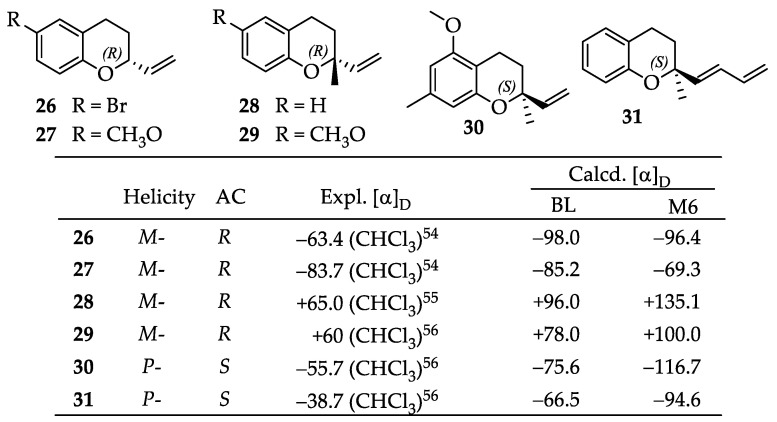
Experimental and predicted SOR values for compounds **26**–**31**. Experimental SOR data were collected from references [54,55,56].

The addition of substituents at the C6 position on the phenyl ring (**18**, **19**, Figure 3, and **26**, **27**, Figure 4) or extending the vinyl group (**15**, Figure 3) exerted little effect on the correlation between the sign of SOR and the C2 stereochemistry of 2-substituted chromanes. Specifically, the presence of an 8-methyl or 8-chloro group might lead to uncertainty in the sign of SOR (Table S1, Entries 41 and 42).

When an additional methyl group was present on the C2 atom (**28**), this methyl group inverted the sign of SOR (Figure 4) [55], which was potentially ascribed to the tremendous difference in the Boltzmann distributions of compounds **24** and **28**. In the conformational search, six stable conformers were identified for both compounds **24** and **28**. However, the Boltzmann distribution of the vinyl groups adopting equatorial and axial positions differed substantially (Table 1).

As shown in Table 1, the vinyl group of **24** was located mainly in the equatorial position, but conformers with the vinyl group located in the axial position were predominant in the conformational mixture of **28**, consistent with the conformational distribution of other 2,2-disubstituted chromanes [28]. Similar to that in compound **28**, the 2-methyl group in compounds **29**–**31** also led to the SOR sign contrary to the correlation prediction.

#### 2.2.4. (*S*)-6-Fluorochromane-2-carboxylic Acid

Chiral chromanes with 2-carboxyl groups, including carboxylic acids and esters, shared the same correlation between SOR and C2 stereochemistry, where isomers with *P*-helicity had positive SORs. Regarding 2-carbonyl chromanes, only two SORs for a pair of enantiomers were retrieved from the literature, which followed this correlation.

**Figure 5:**
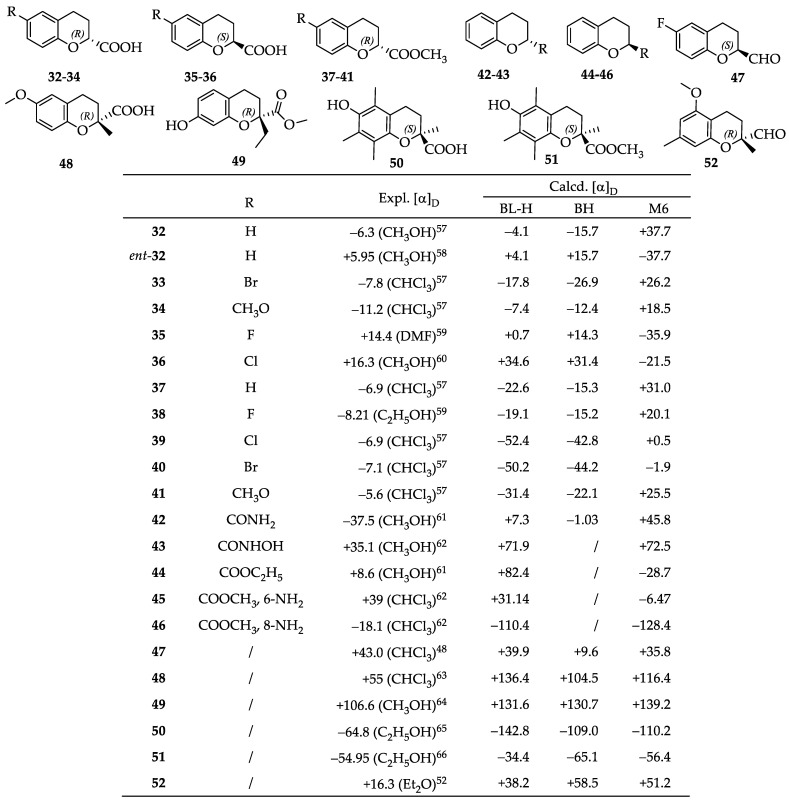
Experimental and predicted [α]D values for compounds **32**–**52**. BL-H: SMD/B3LYP/Aug-cc-pVDZ//B3LYP/6-311+G(d,p), BH: SMD/M06-2X/Aug-cc-pVDZ//BH&HLYP/6-311+G(d,p), M6: SMD/M06-2X/Aug-cc-pVDZ//M06-2X/TZVP. Experimental SOR data were collected from references [48,52,57,58,59,60,61,62,63,64,65,66].

For 2-carboxyl chromanes (**32**–**52**, Figure 5), most of their experimental SORs ranged from −20 to +20, which might be considered unreliable. However, their optical rotation data deduced according to the concentration are high enough to avoid instrument measurement error, so they are included in this study.

Theoretical prediction for compounds with low SORs is often regarded as liable to provide ambiguous results, considering the calculation error of conformational distribution and optical rotation. Nevertheless, since their absolute configurations were firmly established in the original reports, we made an attempt to evaluate the reliability of the calculation. It is particularly important that a reasonable conformational analysis is performed since different conformers might exhibit completely opposite optical rotations. Thus, various computational parameters using different hybrid functionals, basis sets, and solvation models were tested to simulate the actual situation in the conformational mixture.

(*S*)-6-Fluorochromane-2-carboxylic acid (**35**) is commercially available and is the raw material for nebivolol [59]. Generally, optical rotatory dispersion (ORD) is considered more reliable for assigning AC than SOR. Therefore, the experimental ORD of compound **35** was measured in *N,N*-dimethylformamide (DMF), methanol, acetonitrile, and 1,4-dioxane. Specifically, its ${\left[\alpha \right]}_{D}^{27}$ value (+15.2, c 1.0, DMF) was consistent with the reported data (+14.4, c 1.0, DMF) for the *S* isomer [59], indicating that the tested sample had an *S* configuration.

**Figure 6:**
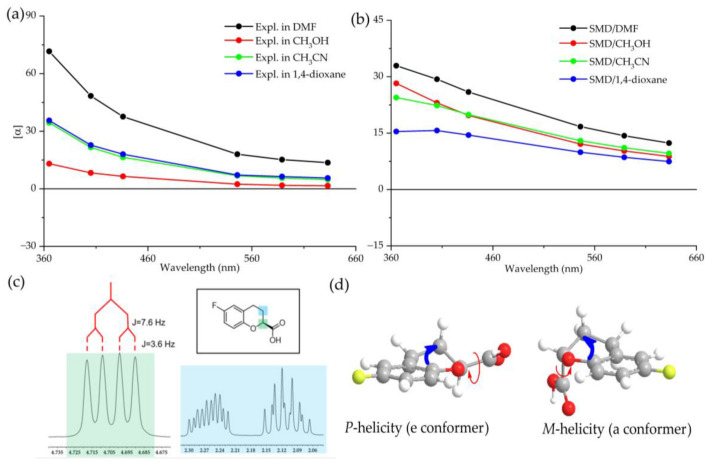
Experimental and theoretical ORD in various solvents and 1H-NMR spectra of **35** in CD3OD. (**a**) Experimental ORD measured in various solvents. (**b**) Theoretical ORD obtained using the SMD/M06-2X/Aug-cc-pVDZ//BH&HLYP/6-311+G(d,p) approach, Black: in DMF, red: in methanol, green: in acetonitrile, blue: in 1,4-dioxane. (**c**) The coupling constants of H2 and two H3 atoms. (**d**) The helicity in the conformers with the carboxyl group in the equatorial and axial positions.

Solvents might exert a slight effect on the conformational distribution of **35**, as the ORD in the tested solvents showed the same tendency with distinct data (Figure 6a), which were repeated by the calculated data (Figure 6b). The solvation effect was also observed in the ECD spectra of **35** in different solvents (Figure S1). However, the ECD spectrum of compound **35** is not as sensitive as that of ORD to solvents. An intense positive Cotton effect (CE) was observed at approximately 230 nm in all the solvents, together with a weak CE at 280 nm, the sign of which varied with the solvent. Thus, for compounds with low SORs, ECD is strongly recommended to study their stereochemistry.

Various computational parameters using different hybrid functionals, basis sets, and solvation models were tested to simulate the actual situation in the conformational mixture of compound **35** (Table S4). Conformers with carboxylic groups in equatorial positions were predominant in the equilibrium mixture, except for in the geometric optimization at the M06-2X/TZVP level. The distribution was consistent with the ^1^H-NMR spectral data of **35**, in which the coupling constant between H2 and two hydrogen atoms at C3 was 7.6 Hz and 3.6 Hz (Figure 6c). Fluctuation of the equatorial/axial position (e/a) ratio resulted in contradictory overall SOR values, and the BH&HLYP (BH) approach yielded SOR data most similar to the experimental [α]_D_ values in DMF. Additionally, the inclusion of a solvent model is indispensable for the SOR calculation for compound **35**. Completely opposite results are obtained when the effect of solvent is neglected and the employment of PCM or SMD provided the correct sign.

As shown for compounds **33**–**47** (Figure 5), esterification or amidation of 2-carboxylic acid (**37**–**42**), halogen (**33**, **35**, **36**, **38**–**40**, **47**), methoxy (**34**, **41**) and amine (**45**) groups at C6 exerted little effect on the correlation (Figure 5). Nevertheless, 2-hydroxamic acid (**43**) or the 8-amine group (**46**) would invert the correlation. As in the cases of compounds **28**–**31**, methyl or ethyl groups at the C2 position (**48**–**52**) would invert the sign of SOR.

For these compounds, the conformational distribution varied tremendously with the computational parameters, resulting in ambiguous calculated SOR results. This situation was particularly evident in chromane-2-carboxylic acids. Among the tested approaches, the BL-H and BH approaches generally tended to provide fair matches with the experimental data with respect to both chroman-2-carboxylic acids and chroman-2-carboxylic esters.

#### 2.2.5. (*S*)-2-Phenylchromane

**Scheme 4:**
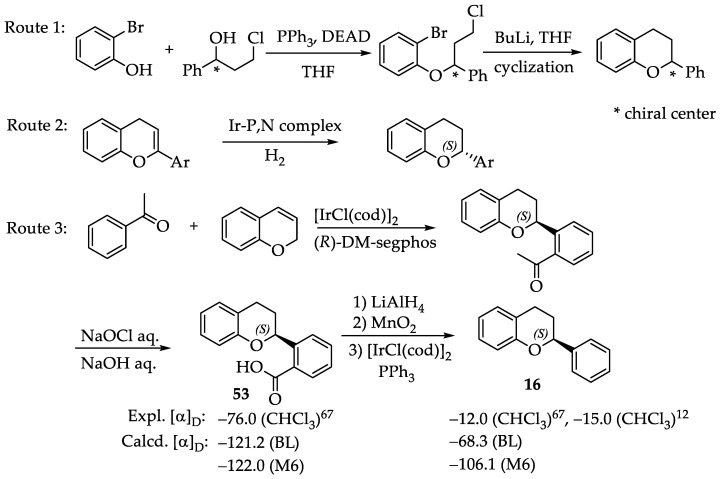
Representative methods for synthesizing chiral 2-aryl chromanes.

Chiral 2-aryl chromanes were obtained using various approaches (Scheme 4), such as a Mitsunobu inversion reaction with subsequent cyclization [11] and iridium-catalyzed asymmetric hydrogenation of chromene [38,67]. Similar to 2-carboxyl (type III) chromanes, many 2-aryl (type II) chromanes display low SORs.

As predicted, compounds **16** and **53** exhibited negative SORs, according to the correlation between the SOR and C2 stereochemistry of 2-substituted chromanes, and this result was further verified by theoretical calculations using the BL- and M6- approaches. An analysis of the conformational distribution of compound **16** showed that the conformer with a 2-phenyl group adopting the equatorial bond accounted for more than 95% of the conformational equilibrium mixture, regardless of the conditions used for geometric optimization. This theoretical result was confirmed by the experimental *J*-couplings of H2 and two H3 atoms (*J* = 10.0, 2.6 Hz) [67].

**Figure 7:**
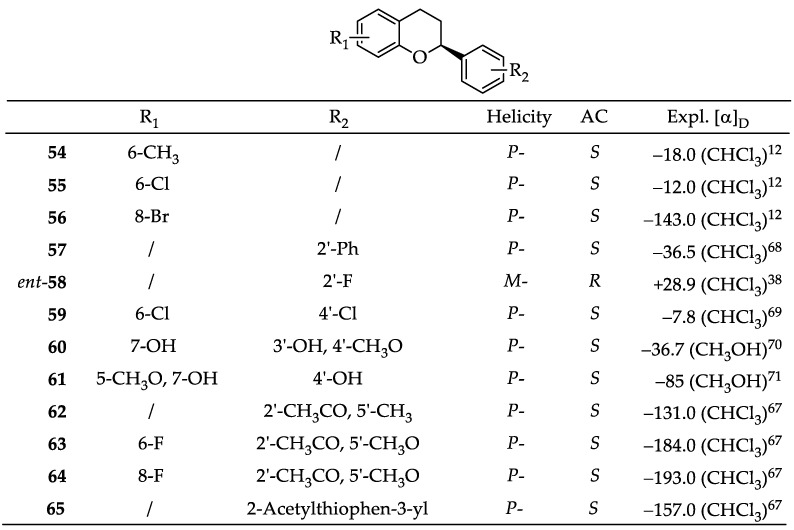
Experimental and predicted [α]_D_ values for chiral 2-aryl chromanes **54**–**65**. Experimental SOR data were collected from references [12,38,67,68,69,70,71].

As exemplified by compounds **54**–**56** (Figure 7), the substituents at either position on the chromane phenyl rings seemed to exert no obvious effect on the SOR sign. Nevertheless, considering the outliers in Figure 1b (Table S2, Entries 5, 11, and 19 with CHO, COOH, and Br at the C6 position), the sum of the electron-donating conjugation effect and electron-absorbing induction effect of groups at the C6 position might play a vital influence on the sign of SOR.

With regard to the 2-phenyl ring, mono- (**17**, **57**, **59**, **61**) or poly- (**60**, **62**–**64**) substitution had little effect on the correlation, even when the substituents were asymmetrically distributed on the phenyl ring. The halogen atom on the C6 or C8 position seemed to increase the absolute value (**63** and **64** vs. **62**).

For 2-aromatic heterocyclic chromanes (**65**), the sign of SOR still complied with the correlation. Nevertheless, more compounds are needed to determine the exact effect of substituents on the aromatic rings.

#### 2.2.6. 1-(6-Fluorochroman-2-yl)ethane-1,2-diol

**Figure 8:**
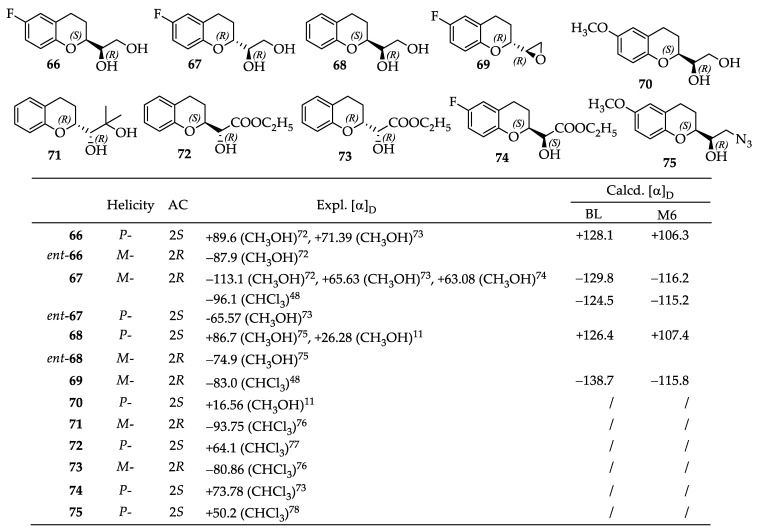
Some reported intermediates of nebivolol analogs **66**–**75**. BL: PCM/B3LYP/Aug-cc-pVDZ//B3LYP/6-311G(d,p), M6: PCM/M06-2X/Aug-cc-pVDZ//M06-2X/TZVP. Experimental SOR data were collected from references [11,48,72,73,74,75,76,77,78].

Two chiral centers exist in this compound and its analogs (Figure 8), and the effect of C2 stereochemistry on SORs deserves further investigation. Within the collected compounds of such kind, C2 chirality seems to be the determining factor for their SORs, and the correlation between SORs and C2 stereochemistry for 2-aliphatic chromanes still works.

The dihydroxy (**66**–**68**, **70**, **71**), carboxylate (**72**–**74**), azide (**75**), and ethylene epoxide (**69**) groups in the C2 substituent and the 6-fluoro (**66**, **67**, **69**, and **74**) or 6-methoxy (**70**, **75**) groups appear to exert minor effects on the SORs of these compounds. The theoretical SORs for compounds **66** and **67** calculated using the BL approach in methanol were +128.1 and −129.8, respectively, concordant with the SOR sign predicted based on the correlation. The AC of compound **67** was further inspected by Snatzke’s method and ECD calculations [71,72,73,74,75,76,77,78,79,80,81]. The positive CE sign at approximately 310 nm verified its (*R*,*R*)-configuration.

### 2.3. Application of the Correlation

**Figure 9:**
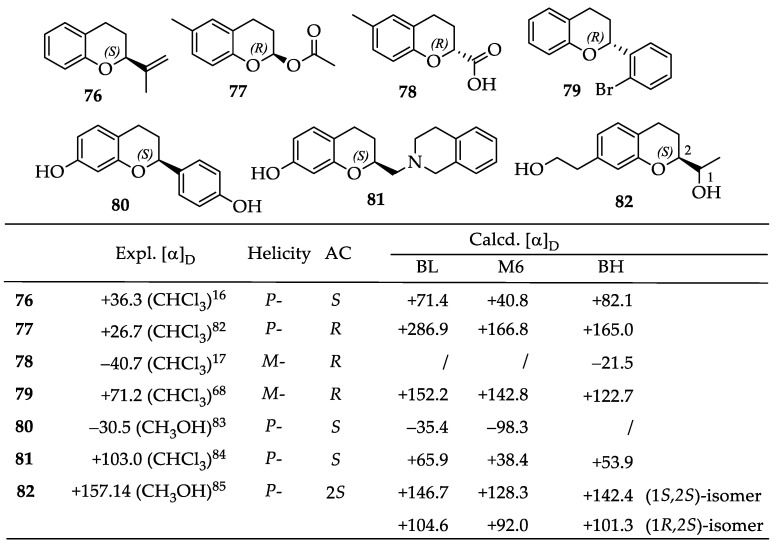
Some chiral 2-substituted chromanes with unidentified configurations. BL: PCM/B3LYP/Aug-cc-pVDZ//B3LYP/6-311G(d,p), M6: PCM/M06-2X/Aug-cc-pVDZ//M06-2X/TZVP, BH: SMD/M06-2X/Aug-cc-pVDZ//BH&HLYP/6-311+G(d,p). Experimental SOR data were collected from references [16,17,68,82,83,84,85].

In this work, a structure-SOR correlation for chiral 2-substituted chromanes was disclosed, with 2-aliphatic and 2-carboxyl chromanes with *P*-helicity displaying positive SORs, while 2-aryl chromanes with *P*-helicity exhibited negative SORs. Then, some chiral 2-substituted chromanes (**76**–**82**, Figure 9) with unknown configurations were studied. Their ACs were tentatively assigned according to the correlations and further confirmed by DFT calculations and mechanistic analyses.

Compound **76** was prepared by catalytic asymmetric cyclization with a chiral ligand, and its AC was not assigned in a previous report [16]. According to the proposed correlation, compound (*S*)-**76** was expected to display a positive SOR, which agreed with the sign of the experimental [α]_D_ value (+36.3, c 1.02, CHCl_3_). This hypothesis was confirmed by the observation that this compound had the same configuration as its analogs that were synthesized via the same route; all were determined to have the *S* configuration by either an X-ray diffraction analysis or a comparison of the retention time obtained from HPLC with that of an authentic sample.

Similar to the structure of compound **12** (Figure 3), an acetoxy group was substituted at the C2 position of compound **77** [82], the *R* isomer of which was predicted to have a positive SOR. The SORs of compound **77** calculated using the BL and M6 approaches were also positive, validating the hypothesis.

Compound **78** was expected to have the *R* configuration based on its [α]_D_ value (−40.7, c 1.0, CHCl_3_) [17]. This hypothesis was confirmed by comparison with the [α]_D_ value of compound **32** (*R* configuration, [α]_D_ −6.3) because of the minor effect of the 6-methyl group. Since the BH approach was shown to be favorable for 2-carboxylic acid chromanes (**32**–**36**), the SOR value of compound **78** calculated using this approach was regarded as more reliable and had the same sign as the predicted SOR value, according to the correlation. The assignment according to the correlation is very convenient and could be performed in minutes by a nonexpert. Nevertheless, the effects of substituents and solvents must be considered in some cases, and further confirmation using alternative, independent methods is necessary whenever crucial.

In the chemical structures of compounds **79** and **80**, substituents are present on the 2-phenyl and chromane benzene rings [68,83]. As above-mentioned, the substituent effect might be neglected, and thus, the ACs of **79** and **80** were directly established as *R* and *S*, respectively, according to the correlation between SOR and C2 stereochemistry.

Despite the presence of the tetrahydroisoquinoline ring in the C2 substituent, compound **81** was viewed as a type I chromane since this ring was connected to the C2 atom through a methylene group [84]. As mentioned above, the effects of substituents on the chromane phenyl ring were often ignored in this type of structure. The AC of compound **81** was thus deduced as *S* since the [α]_D_ value was positive, which was further confirmed by DFT calculations.

Two chiral centers exist in compound **82** [85], and only C2 chirality was the determining factor for the sign of SOR, similar to the results obtained for compounds **66**–**75**. The C2 stereocenter of compound **82** was assigned directly as *S* based on its positive [α]_D_ value according to the correlation. The relative configurations of C1 and C2 were not firmly established, and two epimers were considered. As expected, the SOR calculations of the (1*S*,2*S*) and (1*R*,2*S*) isomers using both the BL and M6 approaches yielded positive values, regardless of the C1 chirality. Because of the flexibility of the two hydroxyethyl groups, more than one hundred stable conformers of compound **82** were considered, and long computation times were needed to obtain the final result.

## 3. Discussions

Although DFT and TDDFT calculations of chiroptical spectroscopy have been regarded as powerful tools for AC assignment of chiral molecules [18], ambiguous results are often encountered. As shown in our previous work and other reports [33,86], the uncertainty mainly arose from the conformational distribution, which might affect the overall SOR values, as well as the ECD and VCD spectra. The correlation of SORs and C2 stereochemistry described in this study has the advantages of being much more facile, less time-consuming, and less dependent on accurate conformational analysis.

Generally, a firm AC assignment of chiral molecules requires exact evidence to achieve unambiguous results, such as chemical correlation, NMR, and various chiroptical methods, including ORD, ECD, and VCD [87,88]. Regarding SOR for structure determination, some problems limit its application, that is, single values, solvent effects, uncertainty of theoretical calculation, etc. [86]. Chiral impurities might interfere with the measurement and provide incorrect data, as shown by a recent example of (+)-frondosin B [89]. Furthermore, the concentration or enantiomeric excess would also affect the test results by forming intermolecular hydrogen bonds or the Horeau effect [90]. Thus, it is not recommended to determine the AC solely relying on the SORs, and another independent method is needed to verify the results.

There are many natural products or synthetic compounds with more chiral centers containing 2-chromane rings. For these compounds, this correlation might not work well because of the complicated influencing factors on the SORs. Similar to Snatzke’s ECD helicity rule, the premise of the proposed correlation is that the larger substituents adopt the equatorial orientation. If not, this correlation would definitely be reverted, as in compound **28** (Figure 4), in which the smaller methyl group was located in the equatorial position instead of the axial position. This preferred conformation could invert the correlation as well as the ECD helicity rule for chromanes, which was challenged in the case of peperobtusin A [91,92].

Additionally, it needs to be noted that the sign of SOR is correlated with the *P*-/*M*-helicity of the dihydropyran ring instead of the *R*/*S* configuration of the C2 atom. The definite *R* or *S* configuration of the C2 atom should be assigned after the helicity is confirmed according to the Cahn-Ingold-Prelog rules.

## 4. Materials and Methods

(*S*)-6-Fluorochromane-2-carboxylic acid (**35**, >97.0% purity) was purchased from Leyan Reagent (Shanghai, China). The ^1^H-NMR spectra of compound **35** were recorded on a Joel ECZ-400S NMR system using CD_3_OD as a deuterated solvent.

### 4.1. Optical Rotation Measurement

Optical rotation measurements were performed on a Rudolph Autopol V automatic digital polarimeter (Rudolph, MA, USA) at 365, 405, 436, 546, 589, and 633 nm at room temperature. A solution of compound **35** prepared at a concentration of 10 mg/mL in methanol, DMF, acetonitrile, or 1,4-dioxane was tested.

### 4.2. ECD Measurement

ECD spectra of compound **35** in methanol, DMF, acetonitrile, or 1,4-dioxane were recorded at room temperature with a path length of 0.1 cm using a Jasco J-815 CD spectrometer (Jasco Inc., Tokyo, Japan).

### 4.3. Computational Details

A conformational search was carried out in the MMFF94 molecular mechanics force field using the MOE software package [93], and all the conformers within an energy window of 10 kcal/mol were regarded as the initial conformations. Geometric optimization and frequency calculations were performed with Gaussian16 RevB.01 [94] to verify the stability and obtain the energies at 298.15 K and a 1 atm pressure. Various theoretical levels were used, including the different combinations of hybrid functionals (B3LYP, M06-2X, BH&HLYP, APFD, Cam-B3LYP, ωB97XD, and O3LYP) and basis sets (6-311G(d,p), 6-311+G(d,p), and TZVP). The dispersion effects and pseudopotential basis sets were used when necessary. The Boltzmann distribution was calculated according to their Gibbs free energies. The SOR calculation step was run in the static limit at the B3LYP/Aug-cc-pVDZ or M06-2X/Aug-cc-pVDZ level. The PCM or SMD model was used to simulate the measurement conditions. The Boltzmann-averaged SORs were obtained using SpecDis 1.71 software [95].

## 5. Conclusions

The three-dimensional structure of a chiral molecule determines its various chiral spectroscopic properties. In this article, the underlying relationship between the C2 stereochemistry of 2-substituted chromanes and their SORs was disclosed through data mining, synthesis mechanism analysis, and DFT calculations.

It was found that the sign of SOR for C2-chiral chromanes was fundamentally due to the helicity of the dihydropyran ring. The 2-aliphatic or 2-carboxyl chromanes with *P*-helicity tend to exhibit positive SORs. Meanwhile, the 2-aryl chromanes with *M*-helicity often display positive SORs. The effects of various substitutions on the chromane core were preliminarily discussed. The additional methyl group at the C2 position could generally invert this correlation, leading to the opposite sign of SORs.

By adopting different combinations of computational parameters, the accuracy of the popular DFT calculation was assessed for these 2-substituted chromanes. For 2-aliphatic or 2-aryl chromanes, the theoretical calculation could produce SOR signs consistent with the experimental results, although there is a certain deviation in the numerical value. Nevertheless, the DFT prediction of SOR for 2-carboxyl chromanes is error-prone, mainly because of the improper estimation of conformational distribution. Considering the cost and reliability of computation, the proposed correlation of SORs and C2 stereochemistry is easy to apply and does not require complex conformational analysis.

It is commonly recognized that versatile methods are needed to guarantee an unambiguous AC assignment of chiral molecules. Combined with other independent ways, we hope the proposed correlation will be helpful for the AC assignment of chiral 2-substituted chromanes, and its reliability will be tested through applications in the future.

## Figures and Tables

**Table 1:** Conformational analysis of compounds **24** and **28** using two approaches.

Comp.	Conf.	Vinyl Arrangement [a]	Helicity	B3LYP/6-311G(d,p)	M06-2X/TZVP
				P (%)	P (%)
24	C1	e	*M*-	48.23	24.42
	C2	e	*M*-	34.15	32.16
	C3	a	*P*-	6.41	30.05
	C4	e	*M*-	5.68	5.85
	C5	a	*P*-	4.52	5.30
	C6	a	*P*-	1.02	2.22
28	C1	a	*P*-	60.06	73.10
	C2	e	*M*-	19.07	10.24
	C3	a	*P*-	7.66	6.88
	C4	a	*P*-	5.28	3.80
	C5	e	*M*-	4.77	3.56
	C6	e	*M*-	3.16	2.41

[a] The vinyl arrangement indicates that vinyl groups occupy the axial or equatorial bond in the corresponding conformers.

## Data Availability

The Cartesian coordinates of conformers of the mentioned compounds could be provided when asked for.

## Supplementary Materials

The following supporting information can be downloaded at: https://www.mdpi.com/article/10.3390/molecules28010439/s1, Table S1: Experimental [α]_D_ values for 2-aliphatic chromanes; Table S2: Experimental [α]_D_ values for 2-aryl chromanes; Table S3: Experimental [α]_D_ values for 2-carboxyl chromanes; Table S4: Conformational analysis of compound **35** at different levels in DMF; Figure S1: Experimental ECD spectra of **35**.
