# Supplementary material for: Stereochemistry of Chiral 2-Substituted Chromanes: Twist of the Dihydropyran Ring and Specific Optical Rotation
Source: Molecules. 2023 Jan 3;28(1):439. doi: 10.3390/molecules28010439 (PMC9823451; doi:10.3390/molecules28010439)
Supplement: Supplementary file 1 [file molecules-28-00439-s001.zip › molecules-2121980-supplementary.pdf]

Supporting Information

Stereochemistry of chiral 2-substituted chromanes: twist of the dihydropyran ring and specific optical rotation

Bei-Bei Yang#, Fan Gao#, Ya-Dong Yang, Ru Wang, Xin Li, Li Li*

Beijing Key Laboratory of Active Substances Discovery and Druggability Evaluation, Institute of Materia Medica, Chinese Academy of Medical Sciences & Peking Union Medical College, Beijing 100050, China

Table of contents

1. Experimental $[\alpha]_D$ values for 2-aliphatic chromanes S2
2. Experimental $[\alpha]_D$ values for 2-aryl chromanes S16
3. Experimental $[\alpha]_D$ values for 2-carboxyl chromanes S44
4. Conformational analysis of compound **35** at different levels in DMF S47
5. Experimental ECD spectra of **35** S48

# B.-B. Yang and F. Gao contributed equally to this work.
* Corresponding author. Tel.: +86-10-63165247, e-mail: annaleelin@imm.ac.cn (L. Li).

Table S1. Experimental $[\alpha]_D$ values for 2-aliphatic chromanes

Entry	Structure	$[\alpha]_D$	Ref.
1	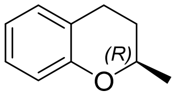	1) +84.1 (0.98, $CHCl_3$), 25°C, ee = 95.7%<br>2) +89.0 (1.0, $CHCl_3$), 22°C	1) Kato Y, Yen D H, Fukudome Y, *et al.* Org. Lett. **2010**, 12(18): 4137-4139.<br>2) Hodgetts K.J. Tetrahedron, **2005**, 61(28): 6860-6870.
2	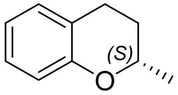	+84.2 (0.47, $CHCl_3$), 26°C, ee = 99.8%	Ma Y, Li J, Ye J, *et al.* Chem. Commun. **2018**, 54(96): 13571-13574.
3	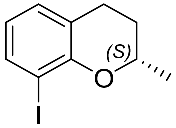	-44.1 (0.32, CH_2Cl_2), 25°C	Birkett M.A, Knight D.W, Little P.B, *et al*. Tetrahedron, **2000**, 56(7): 1013-1023.
4	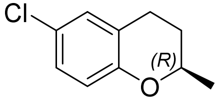	+56.5 (1.7, CH_3OH)	Loiodice F, Longo A, Bianco P, *et al.* Tetrahedron: Asymmetry, **1995**, 6(4): 1001-1011.
5	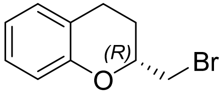	-95.0 (1, CH_3OH)	Gontcharov A. V., Nikitenko A. A., Raveendranath, P, *et al.* WO2007/123941[P]. 2007-11-1
6	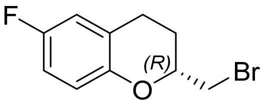	-92.0 (1, CH_3OH)	Gontcharov A. V., Nikitenko A. A., Raveendranath, P, *et al.* WO2007/123941[P]. 2007-11-1
7	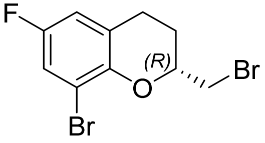	-120.0 (1, CH_3OH)	Gontcharov A. V., Nikitenko A. A., Raveendranath, P, *et al.* WO2007/123941[P]. 2007-11-1
8	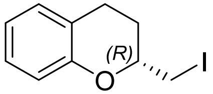	-57.6 (1, $CHCl_3$), 23°C, ee = 90%	Lu Y, Nakatsuji H, Okumura Y, *et al*. J. Am. Chem. Soc. **2018**, 140(19): 6039-6043.

9	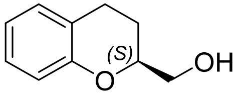	1) +101.2 (0.225, CH_3OH), 20°C<br>2) +87.6 (1.01, CH_3OH), 25°C, ee = 70%<br>3) +90.4 (0.56, CH_3OH), 20°C<br>4) +110.6 (1.55, CH_3OH), 25°C	1) Ida A, Kitao K, Hoshiya N, *et al*. Tetrahedron Lett. **2015**, 56(15): 1956-1959.<br>2) Aponick A, Biannic B. Org. Lett. **2011**, 13(6): 1330-1333.<br>3) Hernández-Torres G, Carreño M.C, Urbano A, *et al*. Eur. J. Org. Chem. **2011**, 2011(20-21): 3864-3877.<br>4) Dinda S.K, Das S.K, Panda G. Synthesis, **2009**, 2009(11): 1886-1896.
10	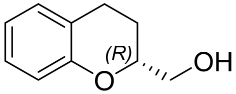	1) -78.5 (1, CH_3OH), 24°C, ee = 90%<br>2) -113.4 (1.12, CH_3OH)	1) Lu Y, Nakatsuji H, Okumura Y, *et al*. J. Am. Chem. Soc. **2018**, 140(19): 6039-6043.<br>2) Urban F.J, Moore B S. J. Heterocycl. Chem. **1992**, 29(2): 431-438.
11	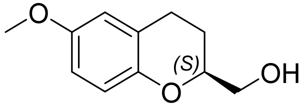	+104.8 (1.5, $CHCl_3$), 25°C, ee = 99%	Dinda S.K, Das S.K, Panda G. Synthesis, **2009**, 2009(11): 1886-1896.
12	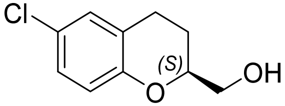	+51.0 (2.5, CH_3OH), 20°C	Loiodice F, Longo A, Bianco P, *et al.* Tetrahedron: Asymmetry, **1995**, 6(4): 1001-1012.
13	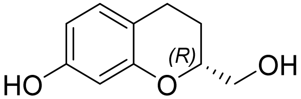	-114.3 (1.01, DMSO), 25°C	Mewshaw R.E, Kavanagh J, Stack G, *et al*. J. Med. Chem. **1997**, 40(26): 4235-4256.
14	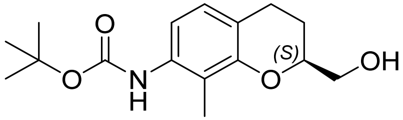	+70.81 (1.0, $CHCl_3$), 25°C	Mewshaw R.E, Marquis K L, Shi X, *et al*. Tetrahedron, **1998**, 54(25): 7081-7108.
15	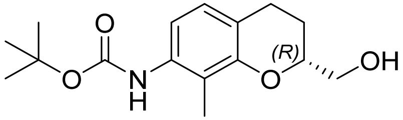	-70.8 (1.0, $CHCl_3$), 25°C	Mewshaw R.E, Marquis K L, Shi X, *et al*. Tetrahedron, **1998**, 54(25): 7081-7108.
16	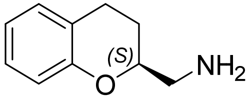	+110.5 (1.0, THF), 20°C	Marco I, Valhondo M, Martín-Fontecha M, *et al*. J. Med. Chem. **2011**, 54(23): 7986-7999.

17	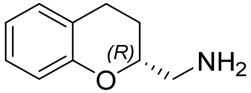	-119.5 (1.0, THF), 20°C	Marco I, Valhondo M, Martín-Fontecha M, *et al*. J. Med. Chem. **2011**, 54(23): 7986-7999.
18	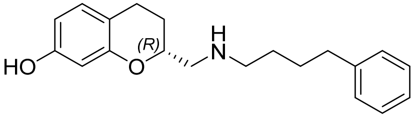	-98.0 (1.4, $CHCl_3$), 25°C	Mewshaw R.E, Kavanagh J, Stack G, *et al*. J. Med. Chem. **1997**, 40(26): 4235-4256.
19	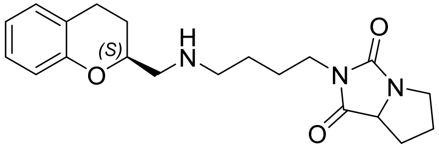	+65.0 (0.5, $CHCl_3$), 20°C	Marco I, Valhondo M, Martín-Fontecha M, *et al*. J. Med. Chem. **2011**, 54(23): 7986-7999.
20	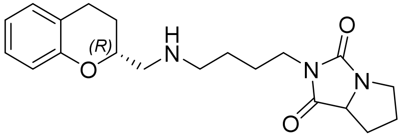	-77.0 (0.5, $CHCl_3$), 20°C	Marco I, Valhondo M, Martín-Fontecha M, *et al*. J. Med. Chem. **2011**, 54(23): 7986-7999.
21	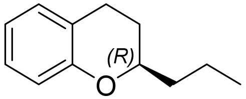	+99.1 (1.96, CH_2Cl_2), 25°C	Cui Y, Villafane L A, Clausen D J, *et al*. Tetrahedron, **2013**, 69(36): 7618-7626.
22	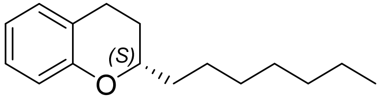	-79.6 (1.36, CH_3OH)	Bonini B.F, Carboni P, Gottarelli G, *et al*. J. Org. Chem. **1994**, 59(20): 5930-5936.
23	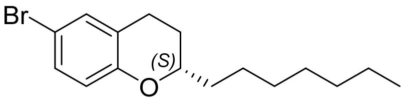	-80.7 (0.84, CH_3OH)	Bonini B.F, Carboni P, Gottarelli G, *et al*. J. Org. Chem. **1994**, 59(20): 5930-5936.
24	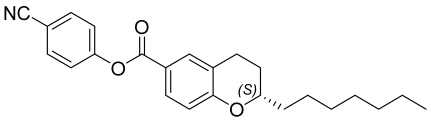	-98.6 (1.19, CH_3OH)	Bonini B.F, Carboni P, Gottarelli G, *et al*. J. Org. Chem. **1994**, 59(20): 5930-5936.
25	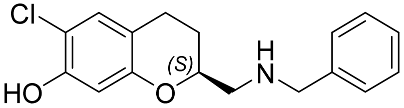	+120.0 (1, $CHCl_3$), 25°C	Mewshaw R.E, Kavanagh J, Stack G, *et al*. J. Med. Chem. **1997**, 40(26): 4235-4256.

26	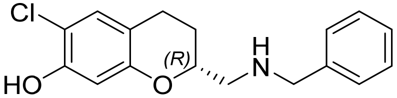	-124.0 (1, $CHCl_3$), 25°C	Mewshaw R.E, Kavanagh J, Stack G, *et al*. J. Med. Chem. **1997**, 40(26): 4235-4256.
27	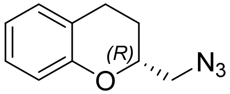	-72.0 (1, CH_3OH)	Gontcharov A. V., Nikitenko A. A., Raveendranath, P, *et al*. WO2007/123941[P]. 2007-11-1
28	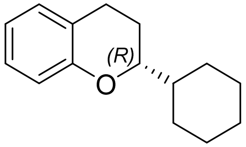	-62.1 (1, $CHCl_3$), 20°C, ee = 95%	Valla C, Baeza A, Menges F, *et al*. Synlett, **2008**, 2008(20): 3167-3171.
29	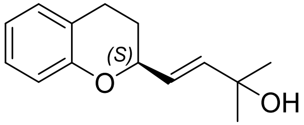	+7.5 (0.4, $CHCl_3$), 25°C	Dinda S.K, Das S.K, Panda G. Synthesis, **2009**, 2009(11): 1886-1896.
30	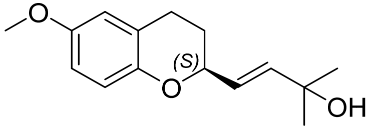	+13.91 (1.75, CH_3OH), 25°C	Dinda S.K, Das S.K, Panda G. Synthesis, **2009**, 2009(11): 1886-1896.
31	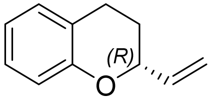	1) -73.8 (1, $CHCl_3$), 25°C, ee = 98%<br>2) -76.5 (0.27, $CHCl_3$), 25°C, ee = 94%<br>3) +80.3 (0.27, CH_2Cl_2), ee = 84%<br>4) -10.3 (1.0, CH_2Cl_2)<br>5) -80.3 (0.27, CH_2Cl_2), ee = 84%	1) Schafroth M A, Rummelt S.M, Sarlah D, *et al*. Org. Lett. **2017**, 19(12): 3235-3238.<br>2) Cannon J.S, Olson A.C, Overman L.E, *et al*. J. Org. Chem. **2012**, 77(4): 1961-1973.<br>3) Trost B.M, Shen H.C, Dong L, *et al*. J. Am. Chem. Soc. **2004**, 126(38): 11966-11983.<br>4) Labrosse J.R, Poncet C, Lhoste P, *et al*. Tetrahedron: Asymmetry, **1999**, 10(6): 1069-1078.<br>5) Trost B.M, Shen H.C, Dong L, *et al.* J. Am. Chem. Soc. **2003**, 125(31): 9276-9277.
32	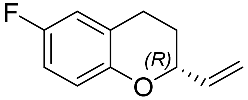	1) -92.9 (0.85, $CHCl_3$), 20°C<br>2) +62.4 (0.35, Et_2O), ee = 80%<br>3) -62.4 (0.35, Et_2O), ee = 80%	1) Carreño M.C, Hernández-Torres G, Urbano A, *et al*. Eur. J. Org. Chem. **2008**, 2008(12): 2035-2038.<br>2) Trost B.M, Shen H.C, Dong L, *et al*. J. Am. Chem. Soc. **2004**, 126(38): 11966-11983.<br>3) Trost B.M, Shen H.C, Dong L, *et al.* J. Am. Chem. Soc.

			**2003**, 125(31): 9276-9277.
33	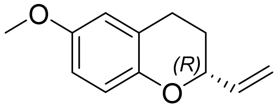	-83.7 (0.54, $CHCl_3$), 25°C, ee = 91%	Cannon J.S, Olson A.C, Overman L.E, *et al*. J. Org. Chem **2012**, 77(4): 1961-1973.
34	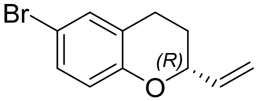	-63.4 (0.4, $CHCl_3$), 25°C, ee = 90%	Cannon J.S, Olson A.C, Overman L.E, *et al*. J. Org. Chem. **2012**, 77(4): 1961-1973.
35	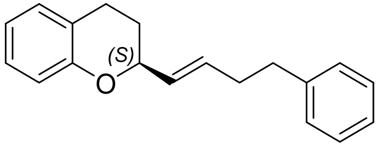	+9.1 (1.2, $CHCl_3$), 23°C	Ida A, Kitao K, Hoshiya N, *et al*. Tetrahedron Lett. **2015**, 56(15): 1956-1959.
36	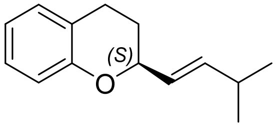	+69.4 (1, CH_2Cl_2), ee = 70%	Aponick A, Biannic B. Org. Lett. **2011**, 13(6): 1330-1333.
37	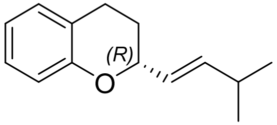	-67.4 (1.0, CH_2Cl_2), ee = 75%	Aponick A, Biannic B. Org. Lett. **2011**, 13(6): 1330-1333.
38	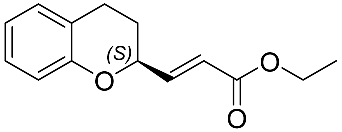	-17.8 (1.46, CH_3OH), 25°C	Dinda S.K, Das S.K, Panda G. Synthesis, **2009**, 2009(11): 1886-1896.
39	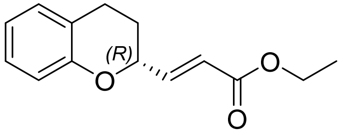	-1.0 (3.0, $CHCl_3$), 20°C	Chung Y.K, Fu G.C. Angew. Chem. Int. Ed. **2009**, 48(12): 2225-2227.
40	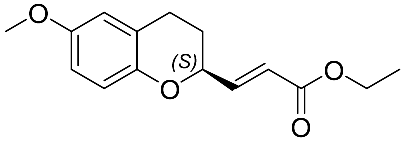	+5.4 (1.21, CH_3OH), 25°C	Dinda S.K, Das S.K, Panda G. Synthesis, **2009**, 2009(11): 1886-1896.

41	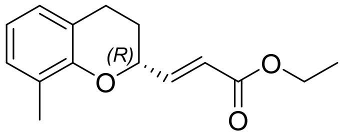	+25.0 (2, $CHCl_3$), 20°C, ee = 84%	Chung Y.K, Fu G.C. Angew. Chem. Int. Ed. **2009**, 48(12): 2225-2227.
42	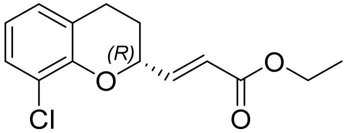	+34.0 (1, $CHCl_3$), 20°C, ee = 63%	Chung Y.K, Fu G.C. Angew. Chem. Int. Ed. **2009**, 48(12): 2225-2227.
43	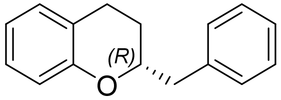	1) -90.0 (1, CH_3OH), 21°C, ee = 88%<br>2) -92.6 (1, $CHCl_3$), 21°C, ee = 88%<br>3) -110.0 (1.0, CH_3OH)	1) Hu N, Li K, Wang Z, *et al.* Angew. Chem. Int. Ed. **2016**, 55(16): 5044-5048.<br>2) Hu N, Li K, Wang Z, *et al.* Angew. Chem. Int. Ed. **2016**, 55(16): 5044-5048.<br>3) Urban F.J, Moore B.S. J. heterocycl. Chem. **1992**, 29(2): 431-438.
44	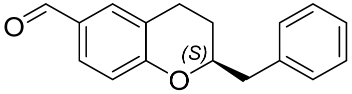	+164.0 (1, CH_3OH)	Urban F.J, Moore B.S. J. heterocycl. Chem. **1992**, 29(2): 431-438.
45	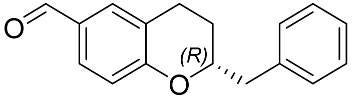	-164.4 (1.0, CH_3OH)	Urban F.J, Moore B.S. J. heterocycl. Chem. **1992**, 29(2): 431-438.
46	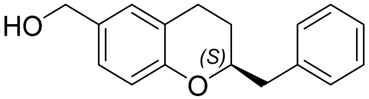	+114.8 (1.0, CH_3OH)	Urban F.J, Moore B.S. J. heterocycl. Chem. **1992**, 29(2): 431-438.
47	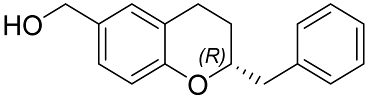	-112.6 (1, CH_3OH)	Urban F.J, Moore B.S. J. heterocycl. Chem. **1992**, 29(2): 431-438.
48	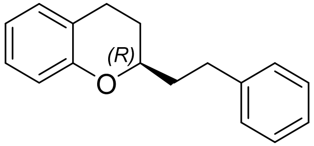	+43.1 (0.8, $CHCl_3$), 24°C, ee = 96%	Srinivas H.D, Maity P, Yap G.P.A, *et al.* J. Org. Chem. **2015**, 80(8): 4003-4016.

49	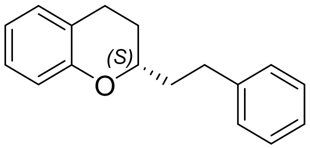	1) -127.7 (0.6, $CHCl_3$), 23°C, ee = 96%<br>2) -103.18 (1.0, $CHCl_3$), 22°C<br>3) -116.3 (1.0, $CHCl_3$), 23°C	1) Guan Y, Attard J.W, Mattson A.E. Chem. Eur. J. **2020**, 26(8): 1742-1747.<br>2) DeRatt L.G, Pappoppula M, Aponick A. Angew. Chem. Int. Ed. **2019**, 58(25): 8416-8420.<br>3) Moquist P.N, Kodama T, Schaus S.E. Angew. Chem. Int. Ed. **2010**, 49(39): 7096-7100.
50	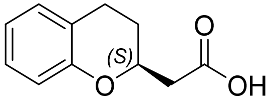	+74.0 (1.56, $CHCl_3$), 26°C, ee = 93%	Azuma T, Murata A, Kobayashi Y, *et al*. Org. lett. **2014**, 16(16): 4256-4259.
51	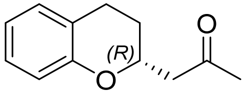	+32.1 (0.47, CH_2Cl_2), 18°C, ee = 36%	Miyaji R, Asano K, Matsubara S. Org. Biomol. Chem. **2014**, 12(1): 119-122.
52	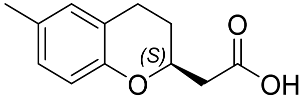	+58.9 (0.58, $CHCl_3$), 26°C, ee = 97%	Azuma T, Murata A, Kobayashi Y, *et al*. Org. Lett. **2014**, 16(16): 4256-4259.
53	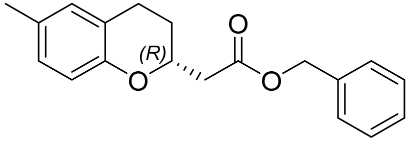	-53.9 (1.04, $CHCl_3$), ee = 92%	Wang L, Yang D, Li D, *et al*. Angew. Chem. Int. Ed. **2018**, 57(29): 9088-9092.
54	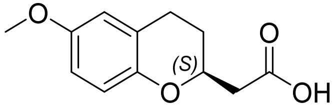	+76.1 (0.74, $CHCl_3$), 25°C, ee = 93%	Azuma T, Murata A, Kobayashi Y, *et al*. Org. Lett. **2014**, 16(16): 4256-4259.
55	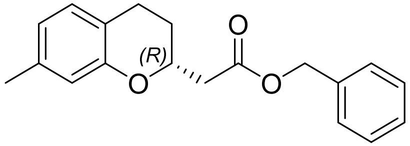	-46.9 (0.96, $CHCl_3$), ee = 92%	Wang L, Yang D, Li D, *et al*. Angew. Chem. Int. Ed. **2018**, 57(29): 9088-9092.
56	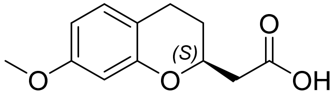	+89.2 (0.89, $CHCl_3$), 25°C, ee = 96%	Azuma T, Murata A, Kobayashi Y, *et al*. Org. Lett. **2014**, 16(16): 4256-4259.

57	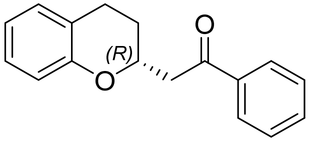	-39.5 (3.61, CH_2Cl_2), 18°C, ee = 84%	Miyaji R, Asano K, Matsubara S. Org. Biomol. Chem. **2014**, 12(1): 119-122.
58	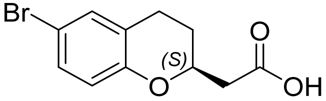	+77.5 (0.98, $CHCl_3$), 26°C, ee = 94%	Azuma T, Murata A, Kobayashi Y, *et al*. Org. Lett. **2014**, 16(16): 4256-4259.
59	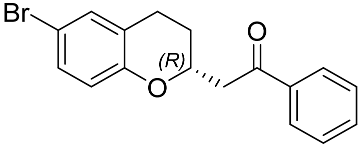	-277.8 (0.17, CH_2Cl_2), 18°C, ee = 65%	Miyaji R, Asano K, Matsubara S. Org. Biomol. Chem. **2014**, 12(1): 119-122.
60	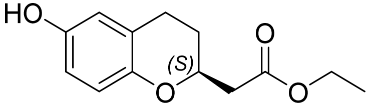	+38.494 (0.35, $CHCl_3$), 21.9°C, ee = 94%	Reddy R.R, Gudup S.S, Ghorai P. Angew. Chem. Int. Ed. **2016**, 55(48): 15115-15119.
61	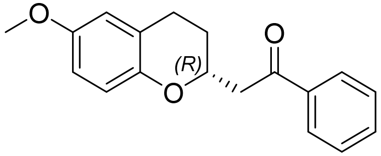	-292.6 (0.54, CH_2Cl_2), 18°C, ee = 74%	Miyaji R, Asano K, Matsubara S. Org. Biomol. Chem. **2014**, 12(1): 119-122.
62	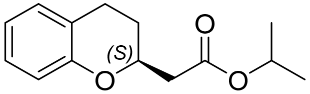	+96.95 (0.5, $CHCl_3$), 20°C	Fischer T, Bamberger J, Gómez-Martínez M, *et al*. Angew. Chem. Int. Ed. **2019**, 58(10): 3217-3221.
63	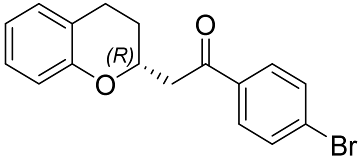	-19.6 (2.8, CH_2Cl_2), 18°C, ee = 83%	Miyaji R, Asano K, Matsubara S. Org. Biomol. Chem. **2014**, 12(1): 119-122.
64	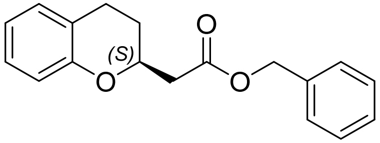	+54.6 (0.3, $CHCl_3$), 25°C	Lee S, Kaib P.S.J, List B. J. Am. Chem. Soc. **2017**, 139(6): 2156-2159.

65	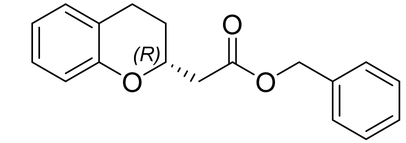	-37.9 (1.03, $CHCl_3$)	Wang L, Yang D, Li D, *et al*. Angew. Chem. Int. Ed. **2018**, 57(29): 9088-9092.
66	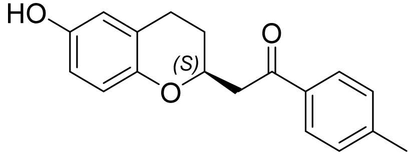	+12.08 (0.45, $CHCl_3$), 20.9°C, ee = 90%	Reddy R.R, Gudup S.S, Ghorai P. Angew. Chem. Int. Ed. **2016**, 55(48): 15115-15119.
67	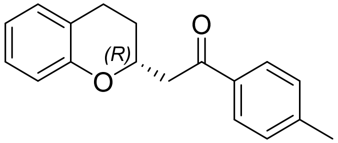	-27.0 (3.95, CH_2Cl_2), 18°C, ee = 84%	Miyaji R, Asano K, Matsubara S. Org. Biomol. Chem. **2014**, 12(1): 119-122.
68	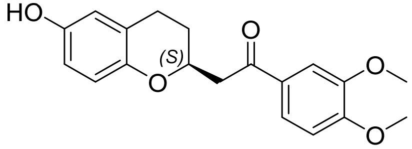	+10.466 (0.3, $CHCl_3$), 21.9°C, ee = 93%	Reddy R.R, Gudup S.S, Ghorai P. Angew. Chem. Int. Ed. **2016**, 55(48): 15115-15119.
69	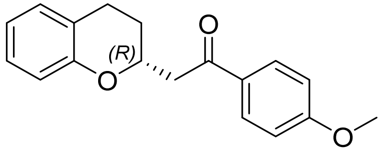	-19.0 (5.2, CH_2Cl_2), 18°C, ee = 84%	Miyaji R, Asano K, Matsubara S. Org. Biomol. Chem. **2014**, 12(1): 119-122.
70	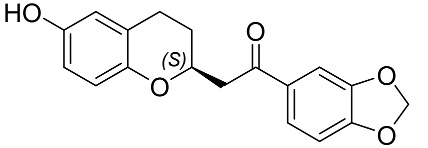	+12.933 (0.15, $CHCl_3$), 22.6°C, ee = 86%	Reddy R.R, Gudup S.S, Ghorai P. Angew. Chem. Int. Ed. **2016**, 55(48): 15115-15119.
71	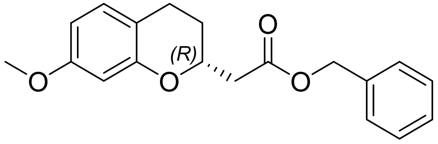	-58.2 (1, $CHCl_3$), ee = 84%	Wang L, Yang D, Li D, *et al*. Angew. Chem. Int. Ed. **2018**, 57(29): 9088-9092.

72	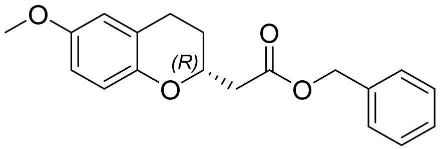	-56.0 (0.98, $CHCl_3$), ee = 93%	Wang L, Yang D, Li D, *et al*. Angew. Chem. Int. Ed. **2018**, 57(29): 9088-9092.
73	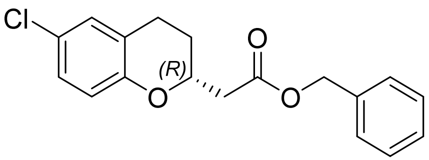	-48.7 (1.01, $CHCl_3$), ee = 74%	Wang L, Yang D, Li D, *et al*. Angew. Chem. Int. Ed. **2018**, 57(29): 9088-9092.
74	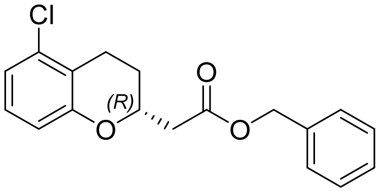	-33.6 (1.01, $CHCl_3$), ee = 77%	Wang L, Yang D, Li D, *et al*. Angew. Chem. Int. Ed. **2018**, 57(29): 9088-9092.
75	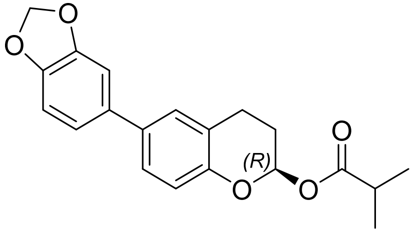	+130.8 (1.47, $CHCl_3$), 25°C, ee = 93%	Glazier D.A, Schroeder J.M, Liu J, *et al*. Adv. Synth. Catal. **2018**, 360(23): 4646-4649.
76	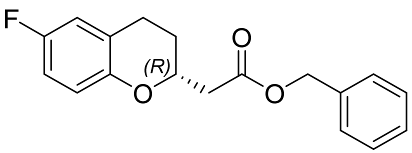	-23.1 (1.0, $CHCl_3$), ee = 86%	Wang L, Yang D, Li D, *et al*. Angew. Chem. Int. Ed. **2018**, 57(29): 9088-9092.
77	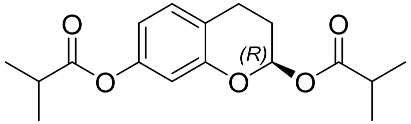	+92.1 (0.635, $CHCl_3$), 25°C, ee = 96%	Glazier D.A, Schroeder J.M, Liu J, *et al*. Adv. Synth. Catal. **2018**, 360(23): 4646-4649.

78	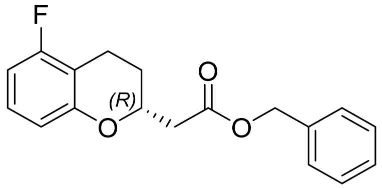	-33.0 (1, $CHCl_3$), ee = 59%	Wang L, Yang D, Li D, *et al*. Angew. Chem. Int. Ed. **2018**, 57(29): 9088-9092.
79	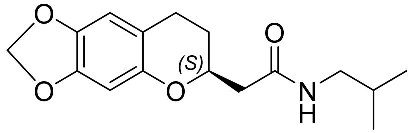	+59.6 (1.81, $CHCl_3$), ee = 94%	Azuma T, Murata A, Kobayashi Y, *et al*. Org. Lett. 2014, 16(16): 4256-4259.
80	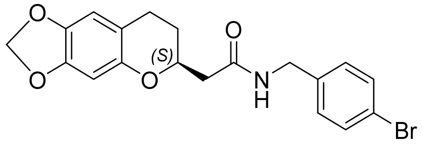	+46.3 (0.47, $CHCl_3$), ee = 91%	Azuma T, Murata A, Kobayashi Y, *et al*. Org. Lett. **2014**, 16(16): 4256-4259.
81	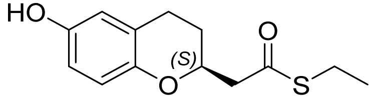	+40.3 (0.2, $CHCl_3$), 28.7°C, ee = 93%	Reddy R.R, Gudup S.S, Ghorai P. Angew. Chem. Int. Ed. **2016**, 55(48): 15115-15119.
82	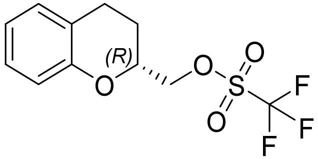	1) -61.9 (1.5, CH_3OH)<br>2) -65.1 (1.0, CH_3OH)	1) Bonini B.F, Carboni P, Gottarelli G, *et al*. J. Org. Chem. **1994**, 59(20): 5930-5936.<br>2) Urban, Moore, J. Heterocycl. Chem. **1992**, 29(2): 431 – 438.
83	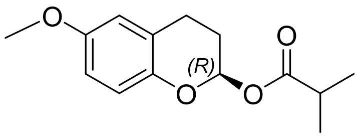	+19.0 (4.3, $CHCl_3$), 25°C, ee = 97%	Glazier D.A, Schroeder J.M, Liu J, *et al*. Adv. Synth. Catal. **2018**, 360(23): 4646-4649.
84	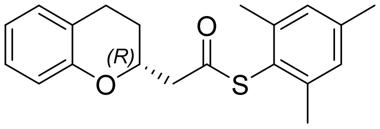	-128.8 (0.4, CH_2Cl_2), 18°C, ee = 75%	Miyaji R, Asano K, Matsubara S. Org. Biomol. Chem. **2014**, 12(1): 119-122.

85	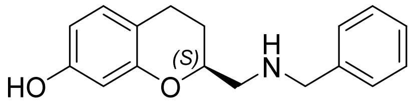	+104.0 (1.15, $CHCl_3$), 25°C	Mewshaw R.E, Kavanagh J, Stack G, *et al*. J. Med. Chem. **1997**, 40(26): 4235-4256.
86	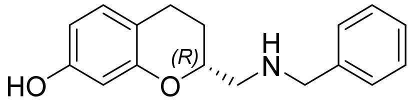	-110.0 (1.04, $CHCl_3$), 25°C	Mewshaw R.E, Kavanagh J, Stack G, *et al*. J. Med. Chem. **1997**, 40(26): 4235-4256.
87	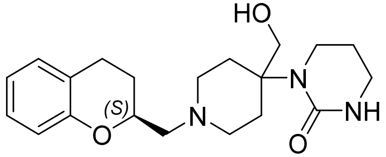	+74.82 (0.052, CH_3OH), 20°C	De Bruyn M.F.L, Van Emelen K, Wigerinck P.T.B P, *et al.* U.S. Patent 6,900,222[P]. 2005-5-31.
88	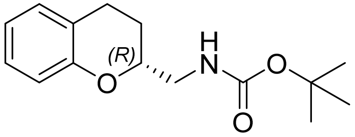	-111.0 (1, CH_3OH)	Gontcharov A. V., Nikitenko A. A., Raveendranath, P, *et al.* WO2007/123941[P]. 2007-11-1
89	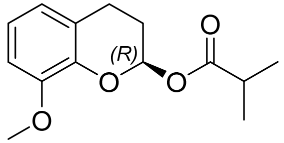	+87.6 (0.94, $CHCl_3$), 25°C, ee = 95%	Glazier D.A, Schroeder J.M, Liu J, *et al*. Adv. Synth. Catal. **2018**, 360(23): 4646-4649.
90	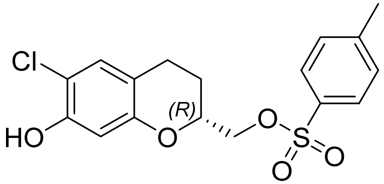	-54.2 (1, $CHCl_3$), 25°C	Mewshaw R.E, Kavanagh J, Stack G, *et al*. J. Med. Chem. **1997**, 40(26): 4235-4256.
91	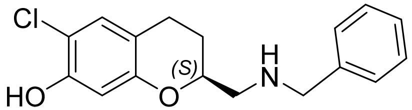	+120.0 (1, $CHCl_3$), 25°C	Mewshaw R.E, Kavanagh J, Stack G, *et al*. J. Med. Chem. **1997**, 40(26): 4235-4256.
92	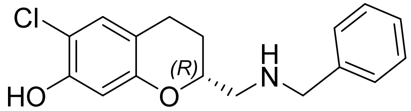	-124.0 (1, $CHCl_3$), 25°C	Mewshaw R.E, Kavanagh J, Stack G, *et al*. J. Med. Chem. **1997**, 40(26): 4235-4256.

93	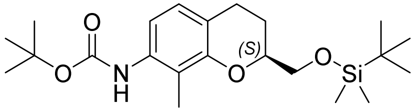	+45.2 (1, $CHCl_3$), 25°C	Mewshaw R.E, Marquis K L, Shi X, *et al*. Tetrahedron, **1998**, 54(25): 7081-7108.
94	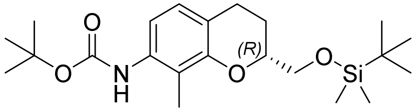	-42.2 (1, $CHCl_3$), 25°C	Mewshaw R.E, Marquis K L, Shi X, *et al*. Tetrahedron, **1998**, 54(25): 7081-7108.
95	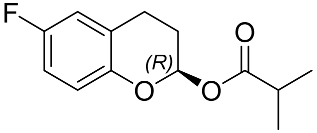	+172.4 (4.6, $CHCl_3$), 25°C, ee = 94%	Glazier D.A, Schroeder J.M, Liu J, *et al*. Adv. Synth. Catal. **2018**, 360(23): 4646-4649.
96	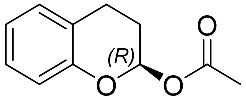	+64.5 (0.2, $CHCl_3$), 20°C, ee = 94%	Gavin D.P, Foley A, Moody T.S, *et al*. Tetrahedron: Asymmetry, **2017**, 28(4): 577-585.
97	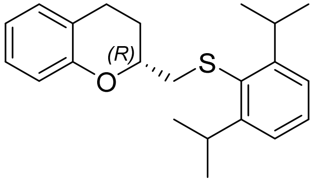	+69.7 (1.01, $CHCl_3$), 23°C	Denmark S.E, Kornfilt D.J.P. J. Org. Chem. **2017**, 82(6): 3192-3222.
98	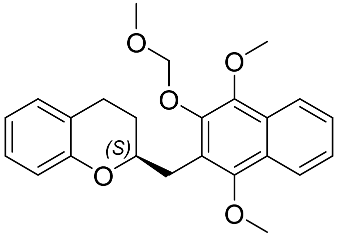	+45.0 (0.92, $CHCl_3$), 20°C	Wakita F, Ando Y, Ohmori K, *et al.* Org. Lett. **2018**, 20(13): 3928-3932.

99	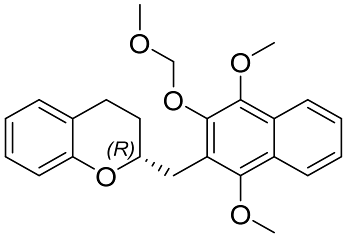	-49.0 (1, $CHCl_3$), 20°C	Wakita F, Ando Y, Ohmori K, *et al.* Org. Lett. **2018**, 20(13): 3928-3932.
100	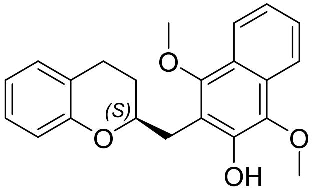	+21.0 (0.99, $CHCl_3$), 20°C	Wakita F, Ando Y, Ohmori K, *et al.* Org. Lett. **2018**, 20(13): 3928-3932.
101	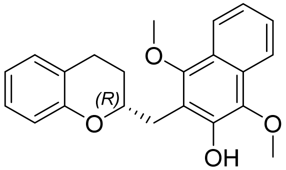	-21.0 (1.05, $CHCl_3$), 20°C	Wakita F, Ando Y, Ohmori K, *et al.* Org. Lett. **2018**, 20(13): 3928-3932.
102	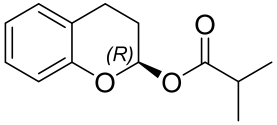	+150.8 (0.01065, $CHCl_3$), 25°C, ee = 96%	Glazier D.A, Schroeder J.M, Liu J, *et al*. Adv. Synth. Catal. **2018**, 360(23): 4646-4649.
103	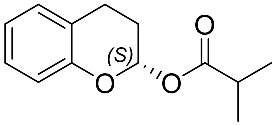	-153.1 (0.00995, $CHCl_3$), 25°C, ee = 96%	Glazier D.A, Schroeder J.M, Liu J, *et al*. Adv. Synth. Catal. **2018**, 360(23): 4646-4649.
104	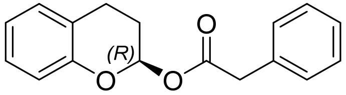	+39.27 (0.62, $CHCl_3$), 25°C, ee = 96%	Glazier D.A, Schroeder J.M, Liu J, *et al*. Adv. Synth. Catal. **2018**, 360(23): 4646-4649.
105	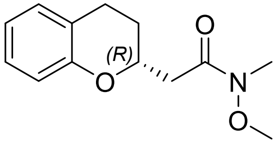	-11.5 (0.35, $CHCl_3$), 25°C	Kobayashi Y, Taniguchi Y, Hayama N, *et al*. Angew. Chem. Int. Ed. **2013**, 52(42): 11114-11118.

106	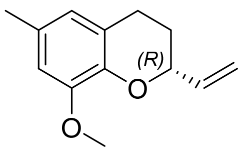	-62.3 (0.21, $CHCl_3$), 82%	Trost B.M, Shen H.C, Dong L, *et al*. J. Am. Chem. Soc. **2004**, 126(38): 11966-11983.

Table S2. Experimental [α]D values for 2-aryl chromanes

Entry	Structure	[α]D	Ref.
1	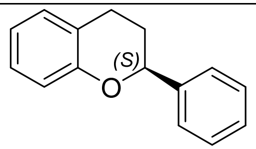	1) -15.0 (0.5, $CHCl_3$), 24°C<br>2) -12.0 (0.6, $CHCl_3$), 25°C, 79%<br>3) -15.0 (3.0, $CHCl_3$), 22°C	1) Antus S, Baitz-Gács E, Kajtár J, *et al.* Liebigs Ann. Chem. **1994**, 1994(5): 497-502.<br>2) Sakamoto K, Nishimura T. Adv. Synth. Catal. **2019**, 361(9): 2124-2128.<br>3) Hodgetts K.J. Tetrahedron, **2005**, 61(28): 6860-6870.
2	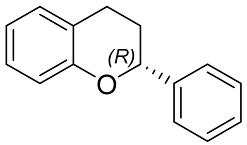	1) +14.4 (1, $CHCl_3$), 27°C, ee = 95%<br>2) +16.6 (1.05, $CHCl_3$), 23°C	1) Lu Y, Nakatsuji H, Okumura Y, et al. J. Am. Chem. Soc. **2018**, 140(19): 6039-6043.<br>2) Denmark S.E, Kornfilt D.J.P. J. Org. Chem. **2017**, 82(6): 3192-3222.
3	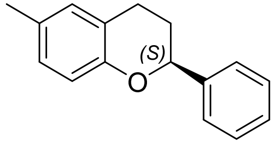	-18.0 (3.0, $CHCl_3$), 22°C	Hodgetts K.J. Tetrahedron, **2005**, 61(28): 6860-6870.
4	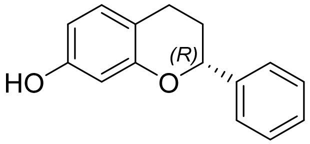	+26.0 (0.48, CHCl3), 20°C	Cardillo G, Merlini L, Nasini G, *et al*. J. Chem. Soc. C: Organic, **1971**: 3967-3970.
5	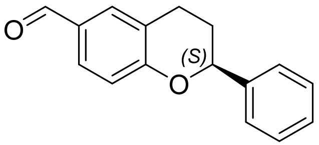	+109.0 (1, $CHCl_3$), 22°C	Hodgetts K.J. Tetrahedron, **2005**, 61(28): 6860-6870.
6	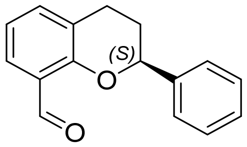	-287.0 (1, $CHCl_3$), 22°C	Hodgetts K.J. Tetrahedron, **2005**, 61(28): 6860-6870.

7	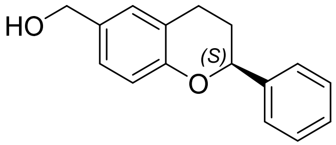	+2.0 (1, $CHCl_3$), 22°C	Hodgetts K.J. Tetrahedron, **2005**, 61(28): 6860-6870.
8	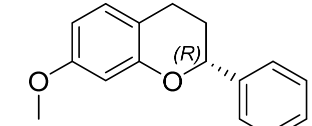	+26.7 (1, $CHCl_3$), 20°C, ee = 99%	Valla C, Baeza A, Menges F, *et al*. Synlett, **2008**, 2008(20): 3167-3171.
9	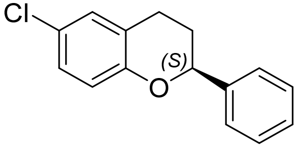	-12.0 (1, $CHCl_3$), 22°C	Hodgetts K.J. Tetrahedron, **2005**, 61(28): 6860-6870.
10	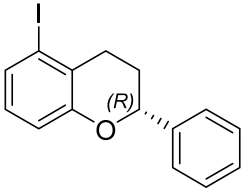	+25.6 (0.36, CH_2Cl_2), 25°C	Birkett M.A, Knight D W, Little P B, *et al*. Tetrahedron, **2000**, 56(7): 1013-1023.
11	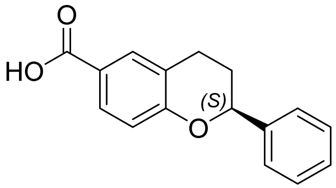	+37.0 (1, $CHCl_3$), 22°C	Hodgetts K.J. Tetrahedron, **2005**, 61(28): 6860-6870.
12	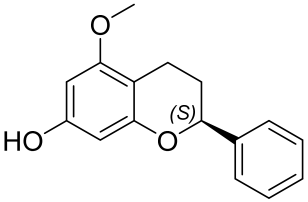	1) -5.3 (0.27, $CHCl_3$)<br>2) -5.7 (0.42, $CHCl_3$), 20°C<br>3) -8.22 (1.08, $CHCl_3$), 24°C	1) Guo Z, Wang Z, Tang Y. Org. Lett. **2018**, 20(7): 1819-1823.<br>2) Schmid M, Trauner D, Angew. Chem. Int. Ed. **2017**, 56(40): 12332 -12335.<br>3) Okamoto A, Ozawa T, Imagawa H, *et al.* Agric. Biol Chem. **1986**, 50(6): 1655-1656.

13	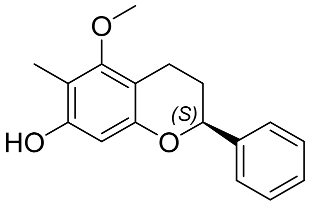	1) -9.3 (2.1, $CHCl_3$), 20°C<br>2) -9.25, 20°C	1) Wang X, Batubara I, Yamauchi K, *et al.* Fitoterapia, **2019**, 138: 104280.<br>2) Cardillo G, Merlini L, Nasini G, et al. J. Chem. Soc. C: Organic, **1971**: 3967-3970.
14	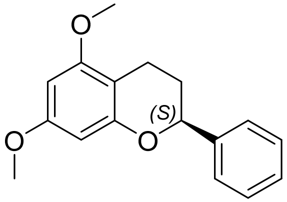	-9.0 (1, $CHCl_3$), 22°C	Hodgetts K.J. Tetrahedron, **2005**, 61(28): 6860-6870.
15	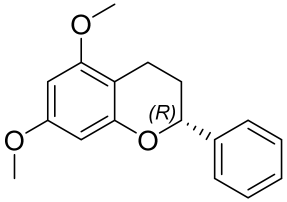	+7.5 (1, $CHCl_3$), 25°C, ee = 96%	Li Y, Wang Z, Ding K. Chem. Eur. J. **2015**, 21(46): 16387-16390.
16	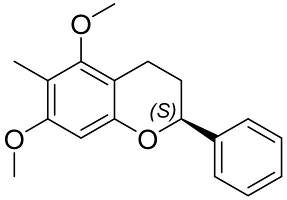	-3.5 (0.3, $CHCl_3$), 20°C	Wang X, Batubara I, Yamauchi K, *et al.* Fitoterapia, **2019**, 138: 104280.
17	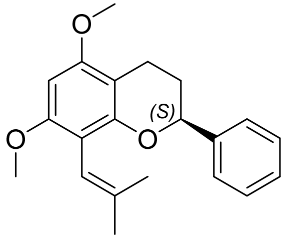	-79.5 (0.088, $CHCl_3$), 20°C	Gomez F, Quijano L, Garcia G, *et al.* Phytochemistry, **1983**, 22(5): 1305-1306.

18	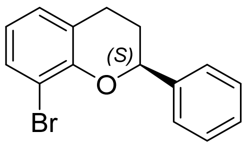	-143.0 (1, $CHCl_3$), 22°C	Hodgetts K.J. Tetrahedron, **2005**, 61(28): 6860-6870.
19	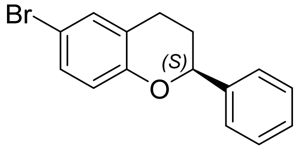	+3.0 (1, $CHCl_3$), 22°C	Hodgetts K.J. Tetrahedron, **2005**, 61(28): 6860-6870.
20	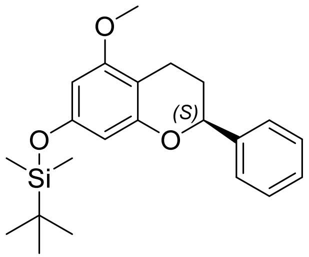	-10.1 (0.375, $CHCl_3$), 22°C	Schmid M, Trauner D. Angew. Chem. Int. Ed. **2017**, 56(40): 12332-12335.
21	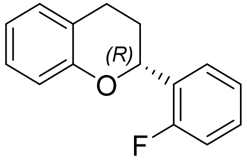	+28.9 (0.5, $CHCl_3$), 20°C, ee = 99%	Valla C, Baeza A, Menges F, *et al*. Synlett, **2008**, 2008(20): 3167-3171.
22	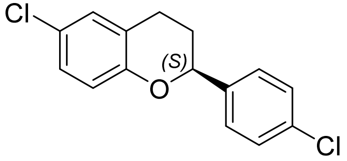	1) -3.2 (1, $CHCl_3$), 20°C<br>2) -7.8 (0.16, $CHCl_3$), 20°C	1) He H, Ye K.Y, Wu Q.F, *et al*. Adv. Synth. Catal. **2012**, 354(6): 1084-1094.<br>2) Choi E.T, Lee M.H, Kim Y, *et al*. Tetrahedron, **2008**, 64(7): 1515-1522.
23	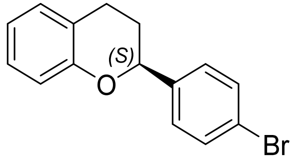	-18.7 (0.5, $CHCl_3$), 20°C, ee = 94%	He H, Ye K.Y, Wu Q.F, *et al*. Adv. Synth. Catal. **2012**, 354(6): 1084-1094.

24	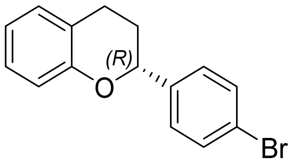	+22.4 (0.5, $CHCl_3$), 20°C, ee = 91%	Valla C, Baeza A, Menges F, *et al*. Synlett, **2008**, 2008(20): 3167-3171.
25	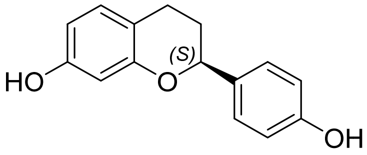	1) -34.5 (0.74, CH_3OH), 27°C<br>2) -23.0 (0.4, CH3OH), 21°C	1) Liu J, Dai H.F, Wu J, *et al*. Zeitschrift für Naturforschung B, **2008**, 63(12): 1407-1410.<br>2) Achenbach H, Stöcker M, Constenla M.A. Phytochemistry, **1988**, 27(6): 1835-1841.
26	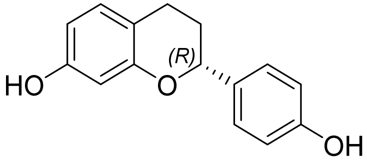	+190.0 (0.04, $CHCl_3$), 24°C	Pan W.B, Chang F.R, Wei L.M, *et al*. J. Nat. Prod. **2003**, 66(2): 161-168.
27	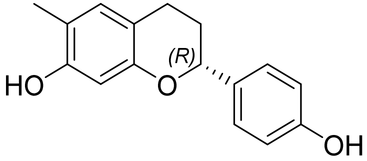	+20.0 (0.3, CH_3OH), 25°C	Li F.X, Wang H, Gai C.J, *et al*. J. Asian Nat. Prod. Res. **2018**, 20(1): 55-61.
28	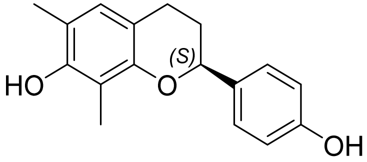	-31.0 (0.58, CH_3OH), 27°C	Liu J, Dai H.F, Wu J, *et al*. Zeitschrift für Naturforschung B, **2008**, 63(12): 1407-1410.
29	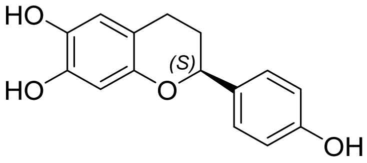	-95.0 (0.25, CH_3OH), 26°C	An R.B, Jeong G.S, Kim Y.C. Chem. Pharm. Bull. **2008**, 56(12): 1722-1724.

30	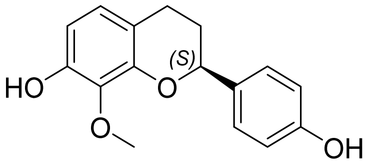	-6.89 (0.58, CH_3OH), 20°C	Camarda L, Merlini L, Nasini G, Heterocycles, 1983, 20(1): 39 – 43.
31	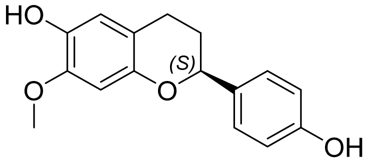	+2.2 (0.51, CH_3OH), 26°C	An R.B, Jeong G.S, Kim Y.C. Chem. Pharm. Bull. **2008**, 56(12): 1722-1724.
32	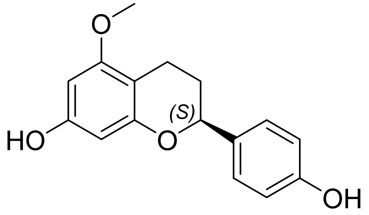	-85.0 (0.1, CH_3OH), 25°C	Nakashima K, Abe N, Kamiya F, *et al*. Helv. Chim. Acta, **2009**, 92(10): 1999-2008.
33	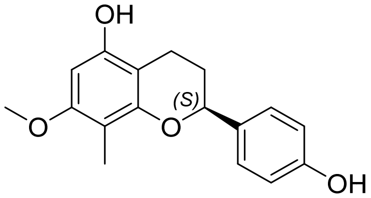	-9.4 (0.489, CH_3OH), 20°C	Camarda L, Merlini L, Nasini G, Heterocycles, 1983, 20(1): 39 – 43.
34	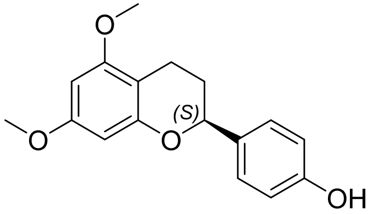	-8.35 (1.16, CH_3OH), 21°C	Okamoto A, Ozawa T, Imagawa H, *et al*. Agric. Biol Chem. **1986**, 50(6): 1655-1656.

35

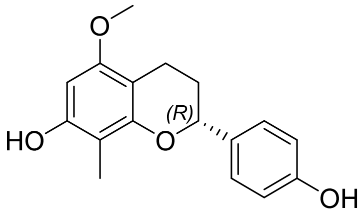

+52.7 (0.19, CH_3OH), 25°C

Awale S, Miyamoto T, Linn T.Z, *et al*. J. Nat. Prod. **2009**, 72(9): 1631-1636.

36

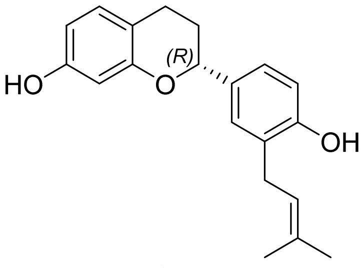

+21.3 (0.08, CH_3OH), 25°C

Xue J.J, Lei C, Wang P.P, *et al*. Fitoterapia, **2018**, 130: 37-42.

37

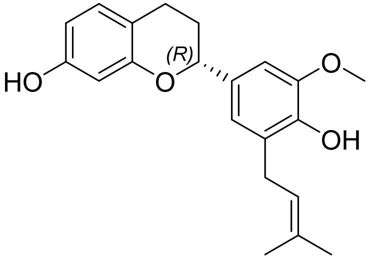

+13.3 (0.04, CH_3OH), 25°C

Xue J.J, Lei C, Wang P.P, *et al*. Fitoterapia, **2018**, 130: 37-42.

38

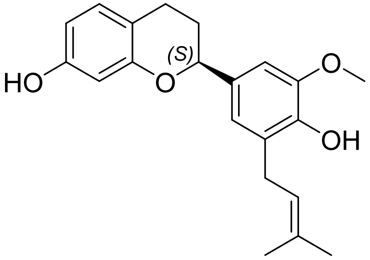

-13.9 (0.04, CH_3OH), 25°C

Xue J.J, Lei C, Wang P.P, *et al*. Fitoterapia, **2018**, 130: 37-42.

39	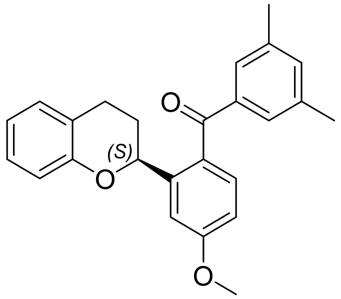	-34.0 (1.48, $CHCl_3$), 25°C	Sakamoto K, Nishimura T. Adv. Synth. Catal. **2019**, 361(9): 2124-2128.
40	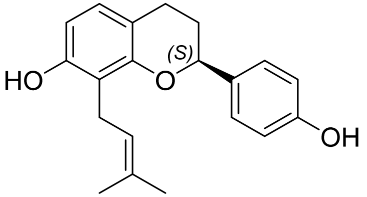	1) -63.4 (0.51, $CHCl_3$), 25°C<br>2) -78.3 (0.59, CH_3OH), 25°C	1) Kessberg A, Metz P. Angew. Chem. Int. Ed. **2016**, 55(3): 1160-1163.<br>2) Kessberg A, Metz P. Angew. Chem. Int. Ed. **2016**, 55(3): 1160-1163.
41	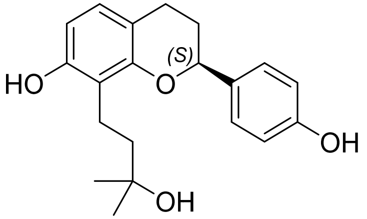	1) -52.4 (0.35, CH_3OH), 25°C, ee = 98%<br>2) -25.3 (0.47, CH_3OH), 22°C	1) Kessberg A, Metz P. Angew. Chem. Int. Ed. **2016**, 55(3): 1160-1163.<br>2) Takashima J, Komiyama K, Ishiyama H, *et al.* Planta Med. **2005**, 71(07): 654-658.
42	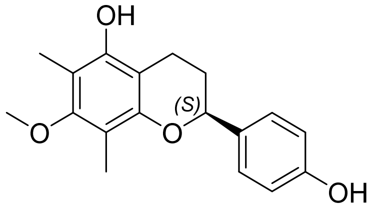	-18.5 (0.5, CH_3OH), 20°C	Chen H.Q, Zuo W.J, Wang H, *et al*. J. Asian Nat. Prod. Res **2012**, 14(5): 436-440.

43	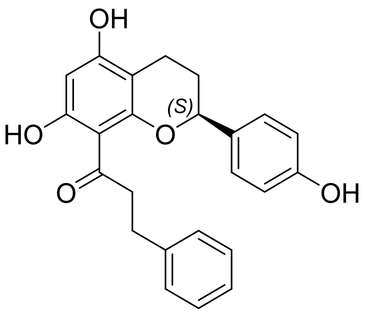	-17.4 (0.25, Acetone), 22°C	Simard F, Legault J, Lavoie S, *et al*. Phytochemistry, **2014**, 100: 141-149.
44	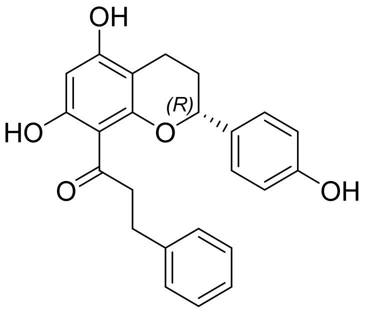	+15.5 (0.16, Acetone), 21°C	Simard F, Legault J, Lavoie S, *et al*. Phytochemistry, **2014**, 100: 141-149.
45	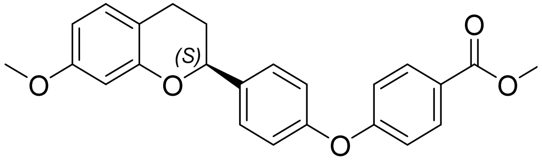	-16.7 (0.59, $CHCl_3$), 25°C	Trinh P.T.N, Tri M.D, Hien D.C, *et al*. Nat. Prod. Res, **2016**, 30(7): 761-767.
46	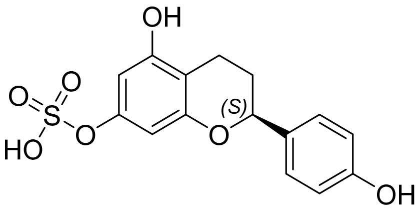	-8.1 (0.09, CH_3OH), 25°C	Karker M, De Tommasi N, Smaoui A, *et al*. Planta Med. **2016**, 82: 1374-1380.

47	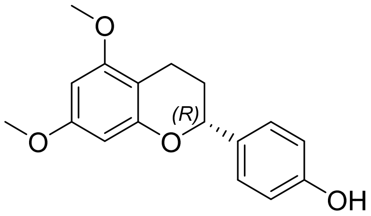	+30.0 (0.15, CH_3OH), 26°C	Siridechakorn I, Cheenpracha S, Ritthiwigrom T, *et al.* Phytochemistry Lett. **2014**, 7: 186-189.
48	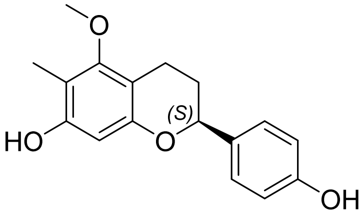	+6.5 (0.2, CH_3CN), 20°C	Wang Y.Y, Dai Y.W, Cao.J, *et al*. Fitoterapia, **2020**: 104549.
49	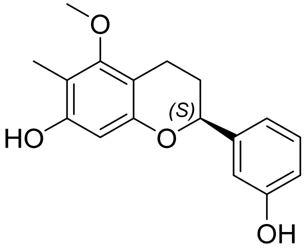	+9.3 (0.15, CH_3CN), 20°C	Wang Y.Y, Dai Y.W, Cao.J, *et al*. Fitoterapia, **2020**: 104549.
50	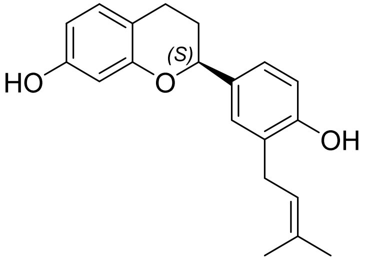	1) -22.0 (0.1, CH_3OH), 25°C<br>2) -4.9 (0.25, CH_3OH), 20°C	1) Xue J.J, Lei C, Wang P.P, *et al*. Fitoterapia, **2018**, 130: 37-42.<br>2) Lee D, Bhat K.P L, Fong H.H.S, *et al*. J. Nat. Prod. **2001**, 64(10): 1286-1293.
51	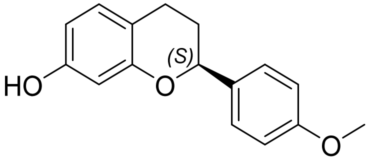	1) -14.7 (0.34, $CHCl_3$), 22°C<br>2) -17.4, $CHCl_3$	1) Ghosal S, Kumar Y, Chakrabarti D.K, *et al.* Phytochemistry, **1986**, 25(5): 1097-1102.<br>2) Takasugi M, Kumagai Y, Nagao S, *et al*. Chem. Lett. **1980**, 9(11): 1459-1460.

52	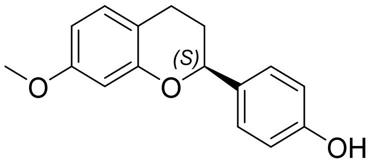	1) -12.0 (0.1, CH_3OH), 26°C<br>2) -16.7 (0.7, CH_3OH), 21°C	1) Min B.S, Gao J.J, Nakamura N, *et al*. Chem. Pharm. Bull. **2001**, 49(9): 1217-1219.<br>2) Achenbach H, Stöcker M, Constenla M.A. Phytochemistry, **1988**, 27(6): 1835-1841.
53	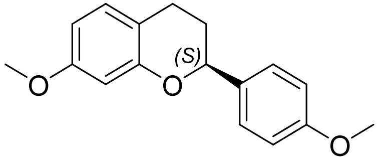	-17.0 (0.1, $CHCl_3$), 25°C	Nhung L.T.H, Linh N.T.T, Cham B.T, *et al*. Nat. Prod. Res. **2019**: 1-8.
54	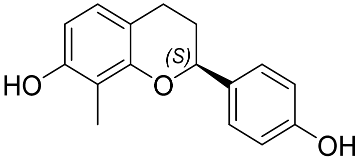	1) -49.2 (0.12, $CHCl_3$)<br>2) +31.6 (0.27, CH_3OH), 20°C	1) Ioset J.R, Marston A, Gupta M.P, *et al*. Fitoterapia, **2001**, 72(1): 35-39.<br>2) Camarda L, Merlini L, Nasini G, Heterocycles, 1983, 20(1): 39 – 43.
55	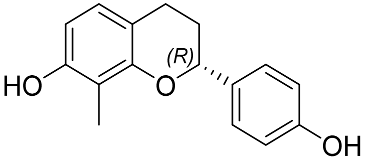	1) +79.2 (0.4, $CHCl_3$), 25°C<br>2) +15.0 (0.41, $CHCl_3$), 27°C	1) Li F.X, Wang H, Gai C.J, *et al*. J. Asian Nat. Prod. Res. **2018**, 20(1): 55-61.<br>2) Liu J, Dai H.F, Wu J, et al. Zeitschrift für Naturforschung B, **2008**, 63(12): 1407-1410.
56	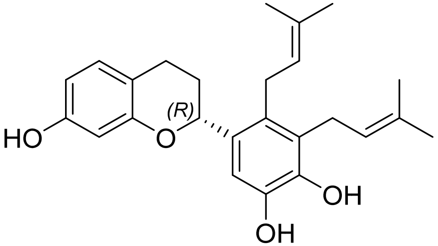	+2.5 (3.05, $CHCl_3$), 28°C	Lee H.J, Lee Y.J, Ryu K.H, *et al*. Bioorg. Med. Chem. Lett. **2010**, 20(12): 3764-3767.
57	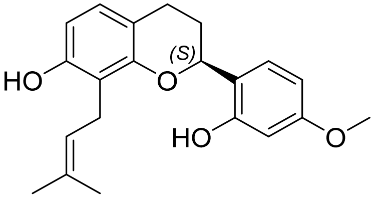	-23.7 (0.1, CH_3OH), 20°C	Park J.H, Lee D.Y, Yun P, *et al*. J. Asian Nat. Prod. Res. **2011**, 13(04): 377-382.

58	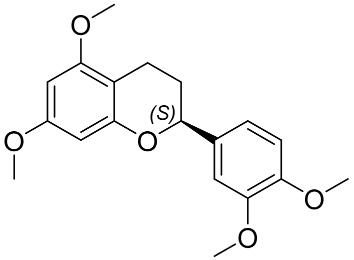	-15.3 (1.0, Acetone), 20°C	Weinges K, Schick H, Zoellner P, Liebigs Annalen der Chemie, **1992**, 3, 293 - 296
59	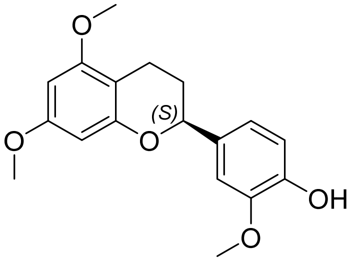	-4.15 (0.25, $CHCl_3$), 23°C	Garo E, Maillard M, Antus S, *et al*. Phytochemistry, **1996**, 43(6): 1265-1269.
60	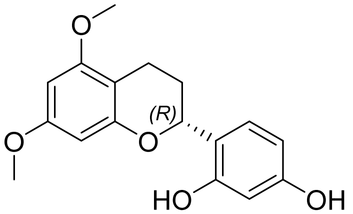	+71.1 (0.15, CH_3OH), 26°C	Siridechakorn I, Cheenpracha S, Ritthiwigrom T, *et al.* Phytochemistry Lett. **2014**, 7: 186-189.
61	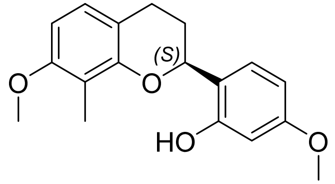	-25.8 (0.7, CH_3OH), 20°C	Tang B.Q, Huang S.S, Liang Y.E, *et al*. Nat. Prod. Res. **2017**, 31(13): 1561-1565.

62	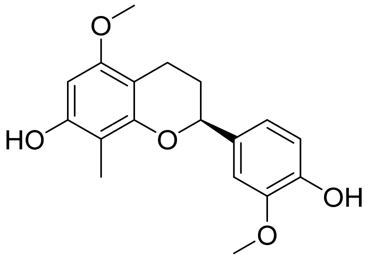	-8.6 (0.1, CH_3OH), 10°C	Zaki A.A, Ross S.A, El-Amier Y.A, *et al*. Phytochemistry Lett. **2018**, 26: 159-163.
63	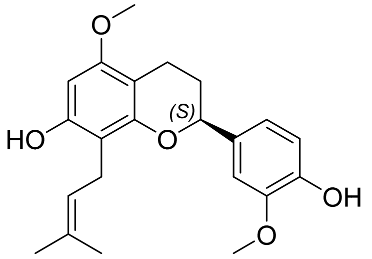	-17.0 (0.1, CH_3OH), 10°C	Zaki A.A, Ross S.A, El-Amier Y.A, *et al*. Phytochemistry Lett. **2018**, 26: 159-163.
64	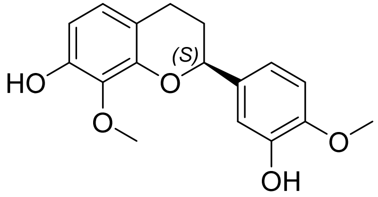	-7.3 (0.1, CH_3OH), 10°C	Zaki A.A, Ross S.A, El-Amier Y.A, *et al*. Phytochemistry Lett. **2018**, 26: 159-163.
65	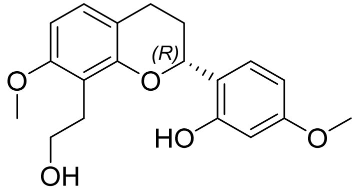	+17.2 (0.1, CH_3OH)	Zhang H.R, Li M, Wang M.M, *et al*. Phytochemistry Lett. **2019**, 29: 84-90.
66	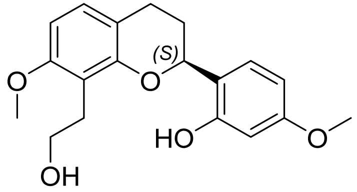	-15.5 (0.1, CH_3OH)	Zhang H.R, Li M, Wang M.M, *et al*. Phytochemistry Lett. **2019**, 29: 84-90.

67	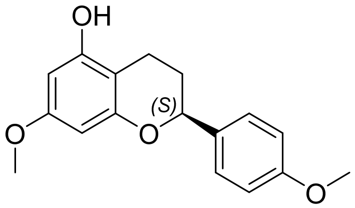	-11.3 (0.34, CH_3OH), 22°C	Ghosal S, Kumar Y, Chakrabarti D.K, *et al*. Phytochemistry, **1986**, 25(5): 1097-1102.
68	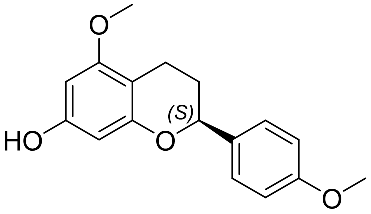	-6.8 (0.47, $CHCl_3$), 22°C	Ghosal S, Kumar Y, Chakrabarti D.K, *et al*. Phytochemistry, **1986**, 25(5): 1097-1102.
69	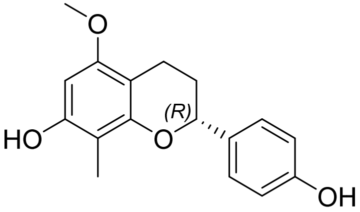	+52.7 (0.19, CH_3OH), 25°C	Awale S, Miyamoto T, Linn T.Z, *et al*. J. Nat. Prod. **2009**, 72(9): 1631-1636.
70	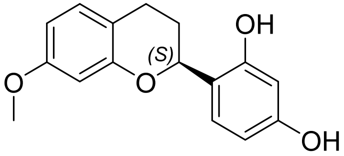	-21.0 (0.1, CH_3OH), 25°C	Nhung L.T.H, Linh N.T.T, Cham B.T, *et al*. Nat. Prod. Res. **2019**: 1-8.
71	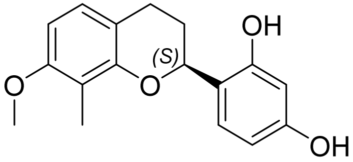	-22.6 (0.8, CH_3OH), 20°C	Tang B.Q, Huang S.S, Liang Y.E, *et al*. Nat. Prod. Res. **2017**, 31(13): 1561-1565.

72	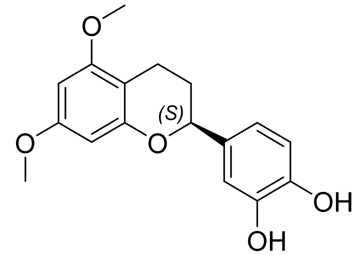	-53.0 (0.36, $CHCl_3$), 20°C	Moosophon P, Kanokmedhakul S, Kanokmedhakul K, *et al*. J. Nat. Prod. **2013**, 76(7): 1298-1302.
73	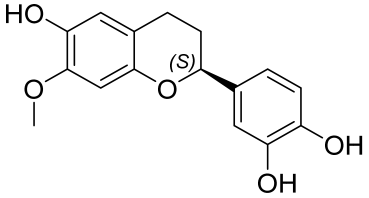	-33.2 (0.52, $CHCl_3$), 20°C	Moosophon P, Kanokmedhakul S, Kanokmedhakul K, *et al*. J. Nat. Prod. **2013**, 76(7): 1298-1302.
74	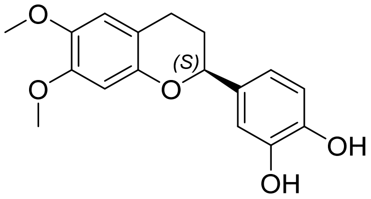	-27.4 (0.37, $CHCl_3$), 20°C	Moosophon P, Kanokmedhakul S, Kanokmedhakul K, *et al*. J. Nat. Prod. **2013**, 76(7): 1298-1302.
75	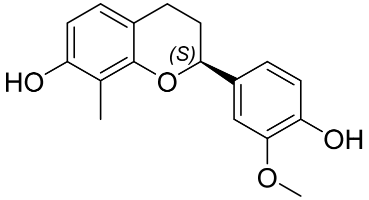	-45.78 (0.68, CH_3OH), 20°C	Camarda L, Merlini L, Nasini G, Heterocycles, 1983, 20(1): 39 – 43.
76	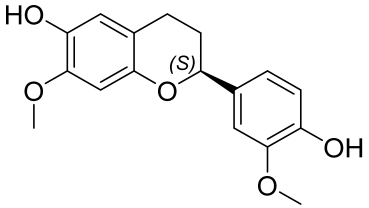	-30.8 (0.27, $CHCl_3$), 20°C	Moosophon P, Kanokmedhakul S, Kanokmedhakul K, *et al*. J. Nat. Prod. **2013**, 76(7): 1298-1302.

77	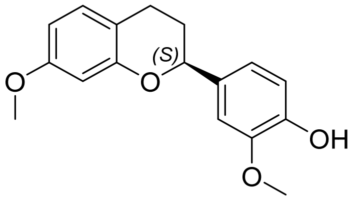	-26.0 (0.13, CH_3OH), 21°C	Achenbach H, Stöcker M, Constenla M.A. Phytochemistry, **1988**, 27(6): 1835-1841.
78	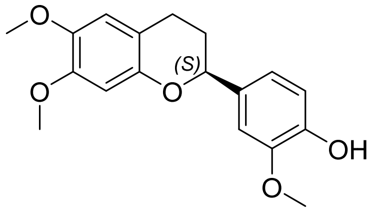	-37.0 (0.2, $CHCl_3$), 20°C	Moosophon P, Kanokmedhakul S, Kanokmedhakul K, *et al*. J. Nat. Prod. **2013**, 76(7): 1298-1302.
79	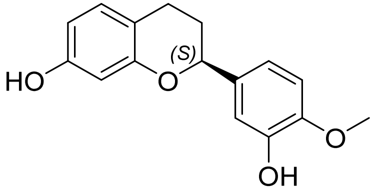	1) -36.7 (0.37, CH_3OH), 25°C, ee = 99%<br>2) -45.5 (0.3, CH_3OH), 24°C	1) Keßberg A, Metz P. Org. Lett. **2016**, 18(24): 6500-6503.<br>2) Masaoud M, Ripperger H, Porzel A, *et al*. Phytochemistry, **1995**, 38(3): 745-749.
80	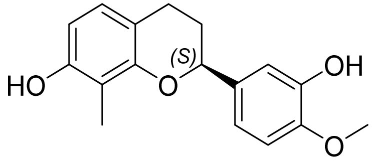	1) -20.0 (0.52, $CHCl_3$), 27°C<br>2) -31.0 (1.44, $CHCl_3$), 20°C	1) Liu J, Dai H.F, Wu J, *et al*. Zeitschrift für Naturforschung B, **2008**, 63(12): 1407-1410.<br>2) Numata A, Takemura T, Ohbayashi H, *et al*. Chem. Pharm. Bull. **1983**, 31(6): 2146-2149.
81	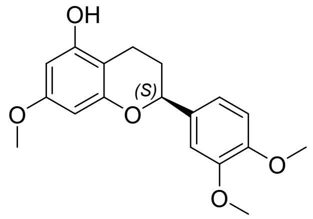	-13.69 (0.16, Acetone), 20°C	Li L.J, Zhang Y, Zhang P, *et al*. J. Asian Nat. Prod. Res. **2011**, 13(04): 367-372.

82	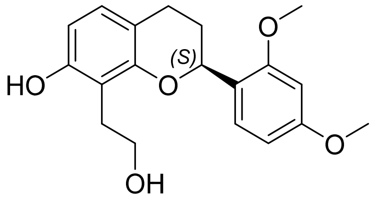	-16.2 (0.07, CH_3OH)	Zhang H.R, Li M, Wang M.M, *et al*. Phytochemistry Lett. **2019**, 29: 84-90.
83	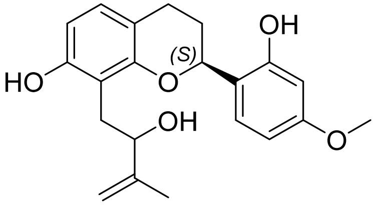	-28.3 (0.1, CH_3OH), 20°C	Zhang H.R, Li M, Wang M.M, *et al*. Phytochemistry Lett. **2019**, 29: 84-90.
84	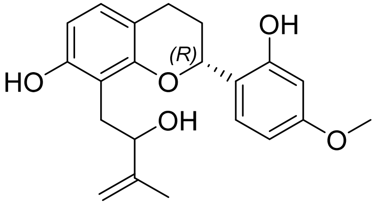	+8.0 (0.1, CH_3OH), 20°C	Zhang H.R, Li M, Wang M.M, *et al*. Phytochemistry Lett. **2019**, 29: 84-90.
85	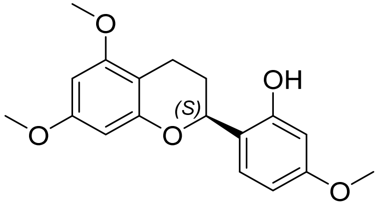	-26.2 (0.9, CH_3OH), 26°C	Morikawa T, Xu F, Matsuda H, *et al*. Chem. Pharm. Bull, **2006**, 54(11): 1530-1534.
86	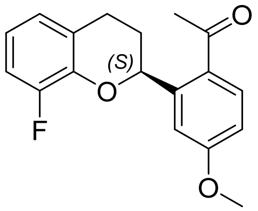	-193.0 (0.5, $CHCl_3$), 25°C, ee = 92%	Sakamoto K, Nishimura T. Adv. Synth. Catal. **2019**, 361(9): 2124-2128.

87	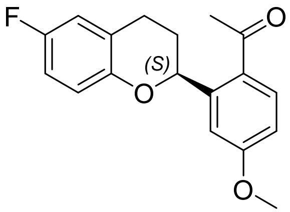	-184.0 (1.57, $CHCl_3$), 25°C, ee = 85%	Sakamoto K, Nishimura T. Adv. Synth. Catal. **2019**, 361(9): 2124-2128.
88	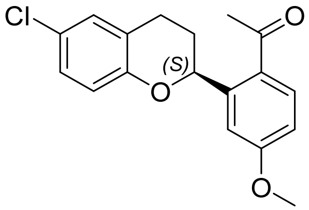	-123.0 (1.1, $CHCl_3$), 25°C, ee = 88%	Sakamoto K, Nishimura T. Adv. Synth. Catal. **2019**, 361(9): 2124-2128.
89	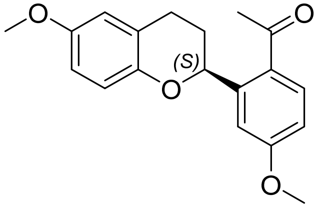	-174.0 (1.2, $CHCl_3$), 25°C, ee = 84%	Sakamoto K, Nishimura T. Adv. Synth. Catal. **2019**, 361(9): 2124-2128.
90	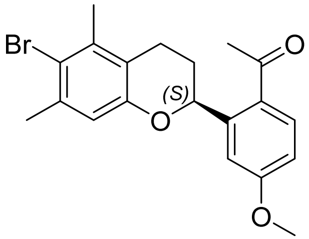	-158.0 (0.89, $CHCl_3$), 25°C, ee = 94%	Sakamoto K, Nishimura T. Adv. Synth. Catal. **2019**, 361(9): 2124-2128.
91	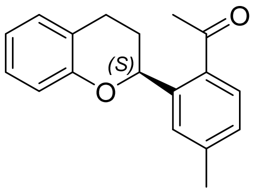	-131.0 (0.74, $CHCl_3$), 25°C, ee = 87%	Sakamoto K, Nishimura T. Adv. Synth. Catal. **2019**, 361(9): 2124-2128.

92	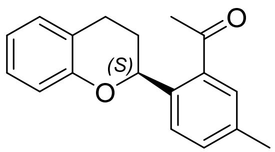	+8.0 (1.16, $CHCl_3$), 25°C, ee = 77%	Sakamoto K, Nishimura T. Adv. Synth. Catal. **2019**, 361(9): 2124-2128.
93	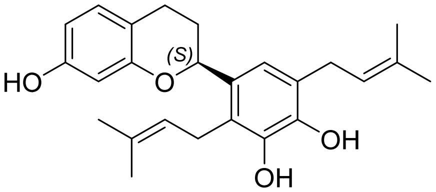	1) -10.7 (0.13, $CHCl_3$)<br>2) -11.0 (0.13, $CHCl_3$), 17°C	1) Park S, Fudhaili A, Oh S.S, *et al*. Phytomedicine, **2016**, 23(12): 1462-1468.<br>2) Ikuta J, Hano Y, Nomura T. Heterocycles (Sendai), **1985**, 23(11): 2835-2842.
94	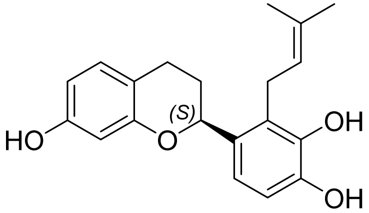	-29.0 (0.1, CH_3OH), 20°C	Sun Q, Yao G.D, Song X.Y, *et al*. Eur. J. Med. Chem. **2017**, 133: 1-10.
95	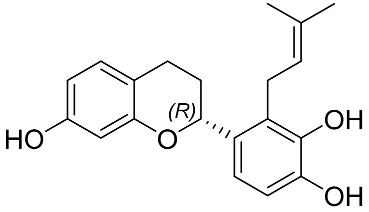	1) +29.0 (0.1, CH_3OH), 20°C<br>2) +4.3 (3.0, CH_3OH), 24°C	1) Sun Q, Yao G.D, Song X.Y, *et al*. Eur. J. Med. Chem. **2017**, 133: 1-10.<br>2) Lee H.J, Lee Y.J, Ryu K.H, et al. Bioorg. Med. Chem. Lett. **2010**, 20(12): 3764-3767.
96	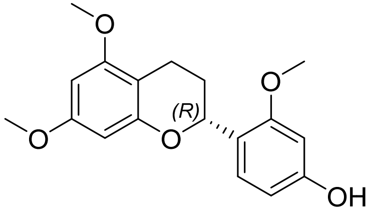	+23.7 (0.23, CH_3OH)	Jiang Z.Y, Bai X.S, Liang H, *et al*. J. Asian Nat. Prod. Res. **2013**, 15(9): 979-984.

97	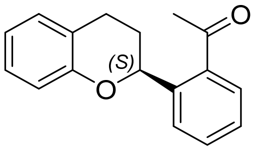	-92.0 (1.08, $CHCl_3$), 20°C, ee = 84%	Ebe Y, Onoda M, Nishimura T, *et al*. Angew. Chem. Int. Ed. 2017, 56(20): 5607-5611.
98	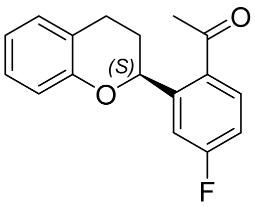	-115.0 (1.49, $CHCl_3$), 20°C, ee = 81%	Sakamoto K, Nishimura T. Adv. Synth. Catal. **2019**, 361(9): 2124-2128.
99	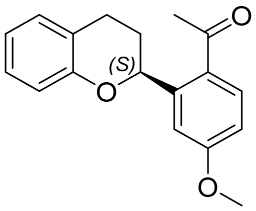	-199.0 (0.98, $CHCl_3$), 25°C	Sakamoto K, Nishimura T. Adv. Synth. Catal. **2019**, 361(9): 2124-2128.
100	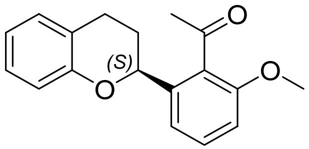	-88.0 (1.14, $CHCl_3$), 25°C	Sakamoto K, Nishimura T. Adv. Synth. Catal. **2019**, 361(9): 2124-2128.
101	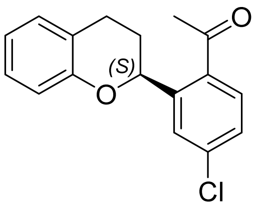	-139.0 (0.62, $CHCl_3$), 25°C	Sakamoto K, Nishimura T. Adv. Synth. Catal. **2019**, 361(9): 2124-2128.

102	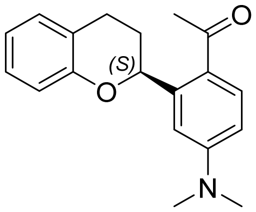	-368.0 (1.42, $CHCl_3$), 25°C, ee = 94%	Sakamoto K, Nishimura T. Adv. Synth. Catal. **2019**, 361(9): 2124-2128.
103	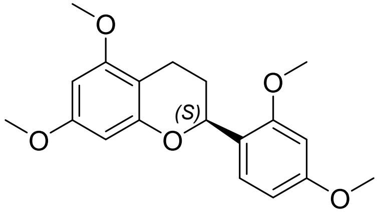	-21.2 (0.4, CH_3OH), 29°C	Morikawa T, Xu F, Matsuda H, *et al*. Chem. Pharm. Bull, **2006**, 54(11): 1530-1534.
104	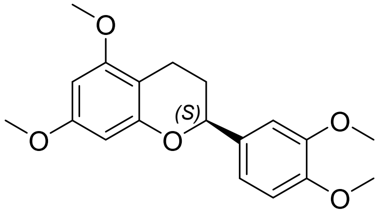	-15.3 (1, Acetone), 20°C	Weinges K, Schick H, Zoellner P, Liebigs Annalen der Chemie, **1992**, 3: 293 - 296
105	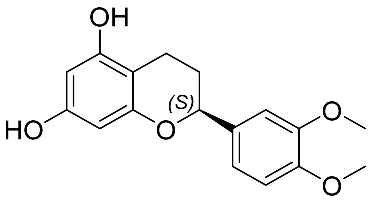	1) -12.4 (0.3, CH_3OH), 22°C<br>2) -10.8 (0.28, CH_3OH), 22°C	1) GHOSAL S, Saini K.S, Sinha B.N. J. Chem. Res. Synopses (Print), **1983** (12): 2601 – 2610.<br>2) GHOSAL S, Saini K.S, Sinha B.N. J. Chem. Res. Synopses (Print), **1983** (12): 2601 – 2610.
106	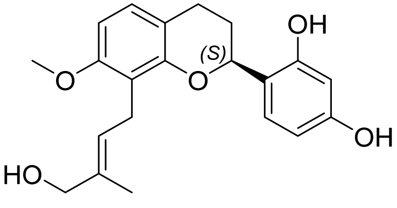	-6.5 (0.6, Acetone), 25°C	Hu X, Wu J.W, Wang M, *et al*. J. Nat. Prod. **2012**, 75(1): 82-87.

107	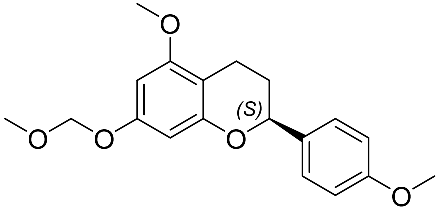	+5.9 (0.31, CH_3CN), 20°C	Yuan H, Bi K, Chang W, *et al*. Tetrahedron, **2014**, 70(47): 9084-9092.
108	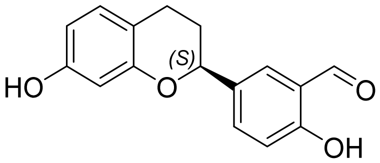	-43.2 (0.12, CH_3OH), 25°C	Sun Q, Li F.F, Wang D, *et al*. RSC Adv. **2016**, 6(61): 55919-55929.
109	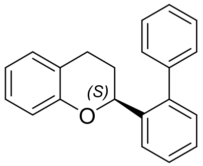	-36.5 (0.4, $CHCl_3$), 20°C, ee = 89%	Wang Y, Franzen R. Synlett, **2012**, 23(06): 925-929.
110	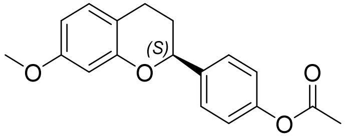	-23.0 (0.2, $CHCl_3$), 21°C	Achenbach H, Stöcker M, Constenla M.A. Phytochemistry, **1988**, 27(6): 1835-1841.
111	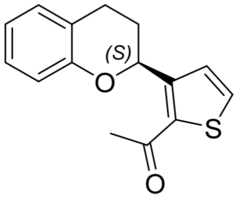	-157.0 (0.91, $CHCl_3$), 25°C, ee = 76%	Sakamoto K, Nishimura T. Adv. Synth. Catal. **2019**, 361(9): 2124-2128.
112	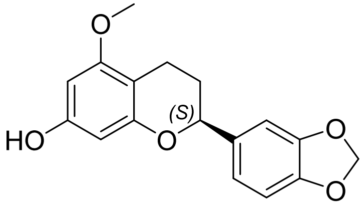	-10.6 (0.5, $CHCl_3$), 20°C	Xu Q, Xie H, Wu P, *et al.* Food chem. **2013**, 139(1-4): 149-154.

113	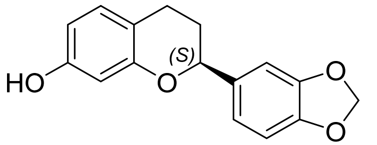	-20.9 (0.2, $CHCl_3$), 24°C	Valsaraj R, Pushpangadan P, Smitt U W, *et al*. J. Nat. Prod. **1997**, 60(7): 739-742.
114	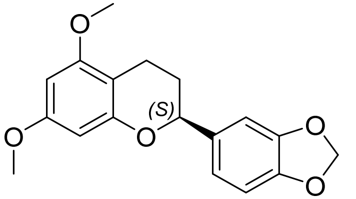	-7.4 (0.5, $CDCl_3$), 25°C	Ho J.C, Chen C.M. Phytochemistry, **2002**, 61(4): 405-408.
115	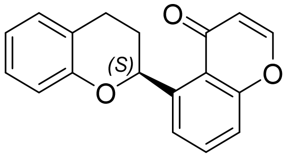	-259.0 (0.89, $CHCl_3$), 25°C, ee = 82%	Sakamoto K, Nishimura T. Adv. Synth. Catal. **2019**, 361(9): 2124-2128.
116	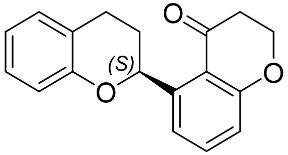	-266.0 (0.74, $CHCl_3$), 25°C, ee = 91%	Sakamoto K, Nishimura T. Adv. Synth. Catal. **2019**, 361(9): 2124-2128.
117	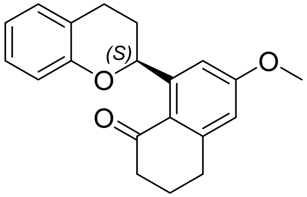	-242.0 (0.91, $CHCl_3$), 25°C, ee = 90%	Sakamoto K, Nishimura T. Adv. Synth. Catal. **2019**, 361(9): 2124-2128.
118	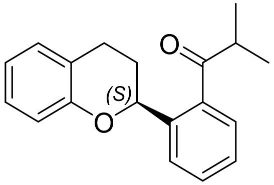	-77.0 (0.53, $CHCl_3$), 25°C, ee = 91%	Sakamoto K, Nishimura T. Adv. Synth. Catal. **2019**, 361(9): 2124-2128.

119	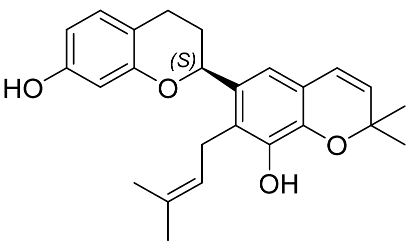	1) -34.8 (0.15, $CHCl_3$), 20°C<br>2) -20.0 (0.38, $CHCl_3$), 24°C	1) Sun Q, Yao G.D, Song X.Y, *et al*. Eur. J. Med. Chem. **2017**, 133: 1-10.<br>2) Ikuta J, Hano Y, Nomura T. Heterocycles (Sendai), 1985, 23(11): 2835-2842.
120	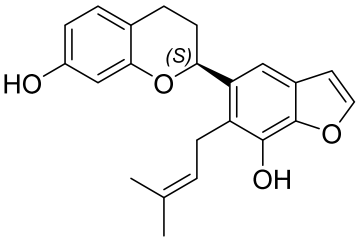	1) -12.2 (0.22, $CHCl_3$), 22°C<br>2) -8.7 (0.1, CH_3OH), 20°C<br>3) -27.5 (0.12, CH_3OH), 25°C	1) Geng C, Chen J. J, Yan M.H, *et al.* CN109172640, **2019**.<br>2) Yang Y.N, An Y.W, Zhan Z.L, *et al*. RSC Adv. 2017, 7(2): 805 - 812.<br>3) Sun Q, Li F.F, Wang D, et al. RSC Adv. **2016**, 6(61): 55919-55929.
121	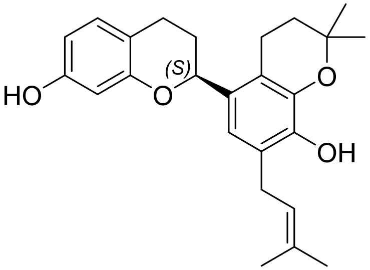	-44.8 (0.1, CH_3OH), 25°C	Sun Q, Li F.F, Wang D, et al. RSC Adv. **2016**, 6(61): 55919-55929.
122	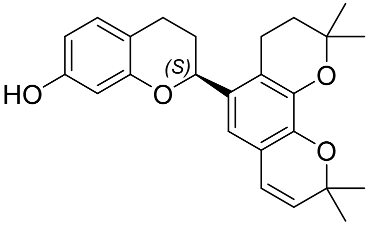	-44.3 (0.11, CH_3OH), 25°C	Sun Q, Li F.F, Wang D, et al. RSC Adv. **2016**, 6(61): 55919-55929.

123	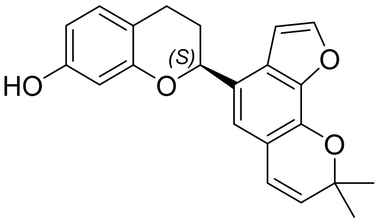	-34.8 (0.07, CH_3OH), 25°C	Sun Q, Li F.F, Wang D, et al. RSC Adv. **2016**, 6(61): 55919-55929.
124	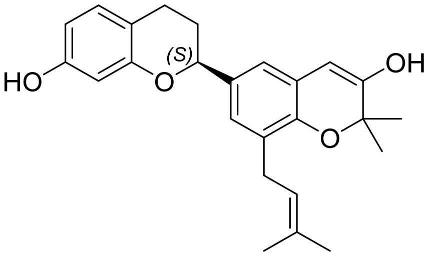	-39.1 (0.09, CH_3OH), 25°C	Sun Q, Li F.F, Wang D, et al. RSC Adv. **2016**, 6(61): 55919-55929.
125	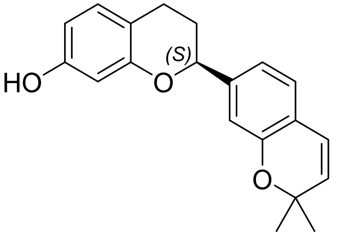	-31.5 (0.13, CH_3OH), 25°C	Sun Q, Li F.F, Wang D, et al. RSC Adv. **2016**, 6(61): 55919-55929.
126	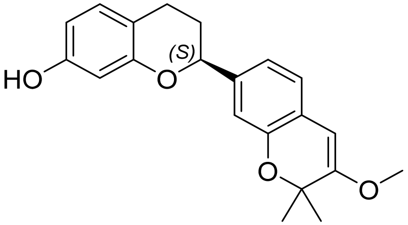	-39.6 (0.05, CH_3OH), 25°C	Sun Q, Li F.F, Wang D, et al. RSC Adv. **2016**, 6(61): 55919-55929.

127	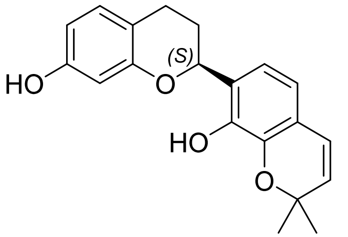	-40.5 (0.08, CH_3OH), 25°C	Sun Q, Li F.F, Wang D, et al. RSC Adv. **2016**, 6(61): 55919-55929.
128	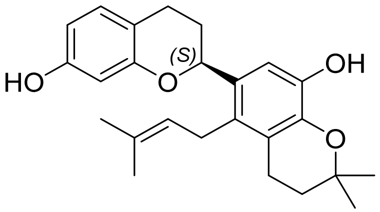	-29.8 (0.1, CH_3OH), 25°C	Sun Q, Li F.F, Wang D, et al. RSC Adv. **2016**, 6(61): 55919-55929.
129	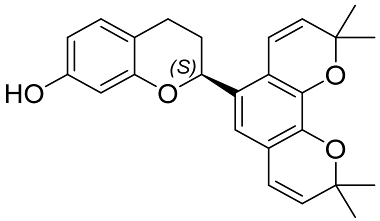	-25.4 (0.09, CH_3OH), 25°C	Sun Q, Li F.F, Wang D, et al. RSC Adv. **2016**, 6(61): 55919-55929.
130	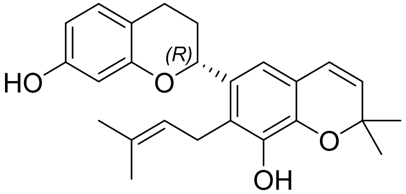	+33.4 (0.08, CH_3OH), 20°C	Sun Q, Yao G.D, Song X.Y, *et al*. Eur. J. Med. Chem. **2017**, 133: 1-10.
131	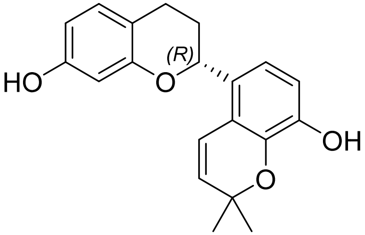	+30.8 (0.08, CH_3OH), 20°C	Sun Q, Yao G.D, Song X.Y, *et al*. Eur. J. Med. Chem. **2017**, 133: 1-10.

132	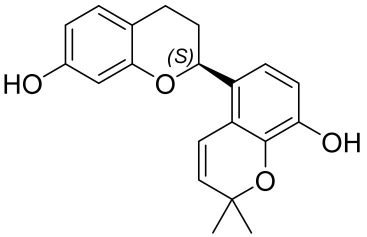	-32.0 (0.09, CH_3OH), 20°C	Sun Q, Yao G.D, Song X.Y, *et al*. Eur. J. Med. Chem. **2017**, 133: 1-10.
133	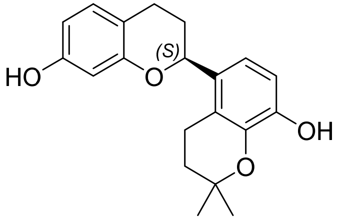	-41.4 (0.06, CH_3OH), 20°C	Sun Q, Yao G.D, Song X.Y, *et al*. Eur. J. Med. Chem. **2017**, 133: 1-10.
134	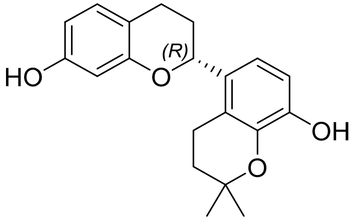	+41.0 (0.06, CH_3OH), 20°C	Sun Q, Yao G.D, Song X.Y, *et al*. Eur. J. Med. Chem. **2017**, 133: 1-10.
135	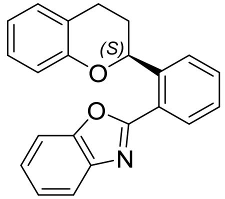	-122.0 (1.01, $CHCl_3$), 20°C	Ebe Y, Onoda M, Nishimura T, *et al*. Angew. Chem. Int. Ed. **2017**, 56(20): 5607-5611.
136	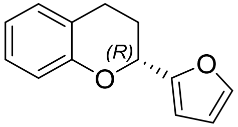	-4.7 (0.9, $CHCl_3$), 20°C, ee = 97%	Valla C, Baeza A, Menges F, *et al*. Synlett, **2008**, 2008(20): 3167-3171.

137	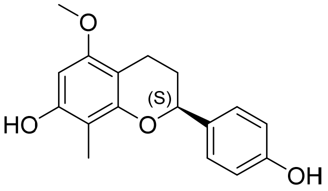	-20.6 (0.1, CH_3OH), 25°C	Lee J. W., Kim J. G. Lee, D. et al. *Phytochem. Lett.* **2021**, *44*, 149-153.
138	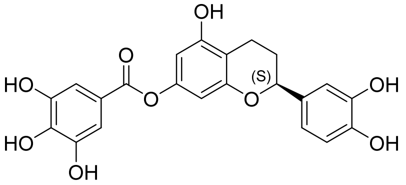	1) -45.3 (0.1, CH_3OH), 23°C<br>2) -26 (0.2, CH_3OH), 25°C	1) Rajachan O. A., Hongtanee L., Chalermsaen K., Kanokmedhakul K., et al. *J. Asian Nat. Prod. Res.* **2020**, *22(5)*, 405-412.<br>2) Jung W. H., Kim K. H., Pang C., et al. *J. Nat. Prod.* **2020**, *83(7)*, 2261-2268.
139	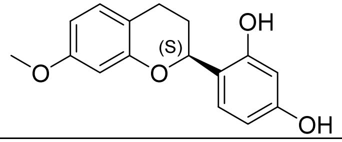	-21 (0.1, CH_3OH), 25°C	Nhung L. T. H., Linh N. T. T., Cham B. T., et al. *Nat. Prod. Res.* **2021**, *35(18)*, 3063-3070.

Table S3. Experimental [α]D values for 2-carboxyl chromanes

Entry	Structure	[α]D	^{1}H NMR data for the H2 atom	Ref.
1	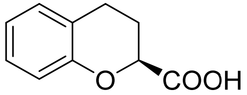	+5.95 (1.058, CH_3OH), 20°C	4.76 (dd, *J* = 6.43, 3.96 Hz, 1H)	Dolle R.E, Chu G.H. U.S. Patent 7,034,051[P]. 2006-4-25.
2	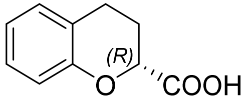	1) -5.97 (1.039, CH_3OH), 20°C<br>2) -6.3 (1.05, CH_3OH), 20°C<br>3) -6.8 (1.0, CH_3OH), 20°C	1) 4.76 (dd, *J* = 6.43, 3.96 Hz, 1H)<br>2) 4.76 (dd, *J* = 7.6, 3.5, 1H)<br>3) Not found	1) Dolle R.E, Chu G.H. U.S. Patent 7,034,051[P]. 2006-4-25.<br>2) Kim D.W, Alam M.M, Lee Y.H, *et al.* Tetrahedron: Asymmetry, **2015**, 26(17): 912-917.<br>3) Shukla M.R, Sarde A.G, Loriya R.M, *et al.* U.S. Patent 9,163,001[P]. 2015-10-20.
3	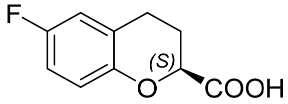	1) +14.4 (1, DMF), 25°C, ee = 95%<br>2) +14.1 (1, DMF), 23°C	1) 4.74 (dd, *J* = 8.1, 3.5 Hz, 1H)<br>2) 4.74 (dd, *J* = 7.8, 3.5 Hz, 1H)	1) Song X.G, Zhu S.F, Xie X.L, *et al.* Angew. Chem. Int. Ed. **2013**, 52(9): 2555-2558.<br>2) Song S, Zhu S.F, Pu L.Y, *et al.* Angew. Chem. Int. Ed. **2013**, 52(23): 6072-6075.
4	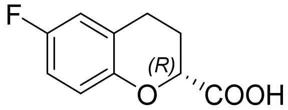	-12.6 (1, DMF), 20°C, ee = 99%	4.74 (dd, *J* = 7.6, 3.5 Hz, 1H)	Kim D.W, Alam M.M, Lee Y.H, *et al.* Tetrahedron: Asymmetry, **2015**, 26(17): 912-917.
5	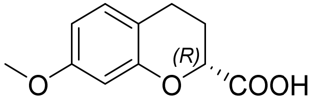	-54.5 (1.25, $CHCl_3$), 20°C, ee = 98%	4.73 (dd, *J* = 7.6, 3.5 Hz, 1H)	Lee Y.S, Lee Y.H, Kim D.W, Patent, KR2017/37154, **2017**.
6	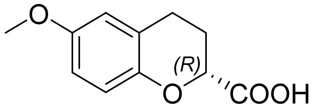	-11.2 (1.35, $CHCl_3$), 20°C, ee = 99%	4.71 (dd, *J* = 7.6, 3.5 Hz, 1H)	Kim D.W, Alam M.M, Lee Y.H, *et al.* Tetrahedron: Asymmetry, **2015**, 26(17): 912-917.
7	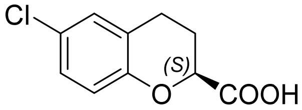	+16.3 (2.1, CH_3OH)	Not found	Loiodice F, Longo A, Bianco P, *et al.* Tetrahedron: Asymmetry, **1995**, 6(4): 1001-1011.

8	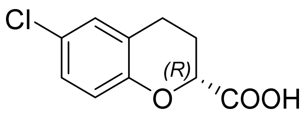	1) -15.5 (1.0, CH_3OH), 20°C, ee = 98%<br>2) -16.4 (2.1, CH_3OH)	1) 4.76 (dd, *J* = 7.6, 3.5 Hz, 1H)<br>2) Not found	1) Kim D.W, Alam M.M, Lee Y.H, et al. Tetrahedron: Asymmetry, **2015**, 26(17): 912-917.<br>2) Loiodice F, Longo A, Bianco P, et al. Tetrahedron: Asymmetry, **1995**, 6(4): 1001-1011.
9	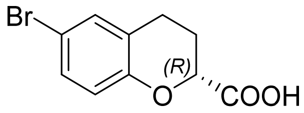	-7.8 (0.5, $CHCl_3$), 20°C, ee = 99%	4.77 (dd, *J* = 7.6, 3.5 Hz, 1H)	Kim D.W, Alam M.M, Lee Y.H, et al. Tetrahedron: Asymmetry, **2015**, 26(17): 912-917.
10	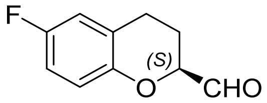	+43.0 (1.4, $CHCl_3$), 20°C	4.44 (dd, *J* = 8.7, 3.3 Hz, 1H)	Carreño M.C, Hernández-Torres G, Urbano A, *et al.* Eur. J. Org. Chem. **2008**, 2008(12): 2035-2038.
11	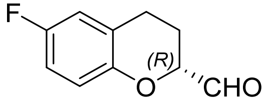	-38.6 (0.53, $CHCl_3$), 20°C	4.44 (dd, *J* = 8.7, 3.3 Hz, 1H)	Carreño M.C, Hernández-Torres G, Urbano A, *et al.* Eur. J. Org. Chem. **2008**, 2008(12): 2035-2038.
12	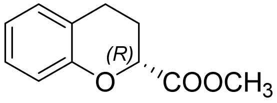	-6.9 (3, $CHCl_3$), 20°C, ee = 99%	4.73 (dd, *J* = 7.6, 3.5 Hz, 1H)	Kim D.W, Alam M.M, Lee Y.H, *et al.* Tetrahedron: Asymmetry, **2015**, 26(17): 912-917.
13	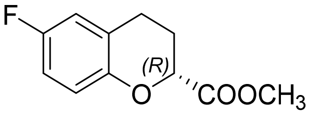	1) -10.3 (0.5, $CHCl_3$), 20°C, ee = 99%<br>2) -8.21 (3.8, EtOH), 25°C, ee = 96%	1) 4.70 (dd, *J* = 7.6, 3.5 Hz, 1H)<br>2) 4.69 (dd, *J* = 7.3, 3.7 Hz, 1H)	1) Kim D.W, Alam M.M, Lee Y.H, et al. Tetrahedron: Asymmetry, **2015**, 26(17): 912-917.<br>2) Song X.G, Zhu S.F, Xie X.L, *et al.* Angew. Chem. Int. Ed. **2013**, 52(9): 2555-2558.
14	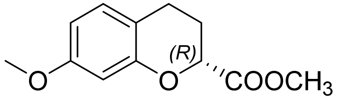	+11.6 (1.8, $CHCl_3$), 20°C, ee = 99%	4.71 (dd, *J* = 7.6, 3.5 Hz, 1H)	Kim D.W, Alam M.M, Lee Y.H, *et al.* Tetrahedron: Asymmetry, **2015**, 26(17): 912-917.
15	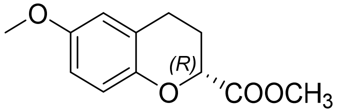	-5.6 (0.2, $CHCl_3$), 20°C, ee = 99%	4.68 (dd, *J* = 7.6, 3.5 Hz, 1H)	Kim D.W, Alam M.M, Lee Y.H, *et al.* Tetrahedron: Asymmetry, **2015**, 26(17): 912-917.

16	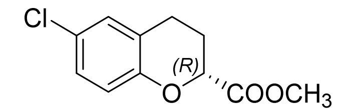	-6.9 (3, $CHCl_3$), 20°C, ee = 99%	4.73 (dd, *J* = 7.6, 3.5 Hz, 1H)	Kim D.W, Alam M.M, Lee Y.H, *et al*. Tetrahedron: Asymmetry, **2015**, 26(17): 912-917.
17	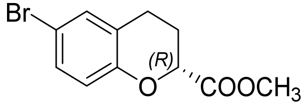	-7.1 (0.5, $CHCl_3$), 20°C, ee = 98%	4.73 (dd, *J* = 7.6, 3.5 Hz, 1H)	Kim D.W, Alam M.M, Lee Y.H, *et al*. Tetrahedron: Asymmetry, **2015**, 26(17): 912-917.
18	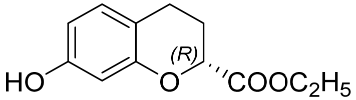	1) -20.1 (1, $CHCl_3$)<br>2) -20.2 (1, $CHCl_3$), 25°C	1) Not found<br>2) Not found	1) van Wieringen J.P, Shalgunov V, Janssen H.M, *et al*. J. Med. Chem. **2014**, 57(2): 391-410.<br>2) Kalaritis P, Regenye R.W, Partridge J.J, *et al*. J. Org. Chem. **1990**, 55(3): 812-815.
19	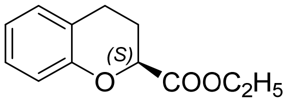	+8.6 (1.24, CH_3OH), 20°C, ee = 95%	Not found	Marco I, Valhondo M, Martín-Fontecha M, *et al*. J. Med. Chem. **2011**, 54(23): 7986-7999.
20	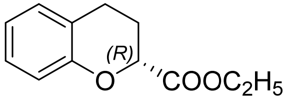	1) -8.7 (1.24, CH_3OH), 20°C, ee = 97%<br>2) -9.3 (1.24, CH_3OH)	1) Not found<br>2) 4.15 (m, 1H)	1) Marco I, Valhondo M, Martín-Fontecha M, et al. J. Med. Chem. **2011**, 54(23): 7986-7999.<br>2) Urban F.J, Moore B S. J. Heterocycl. Chem. **1992**, 29(2): 431-438.
21	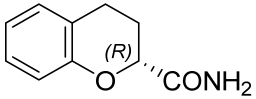	-37.5 (1.2, CH_3OH), 20°C	Not found	Marco I, Valhondo M, Martín-Fontecha M, *et al*. J. Med. Chem. **2011**, 54(23): 7986-7999.
22	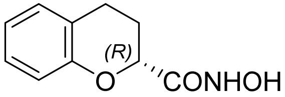	+35.1 (0.24, CH_3OH)	Not found	Wipf, P.; Huryn, D. M.; Laporte, M. G.; et al. **2022**, WO2022/120048.
23	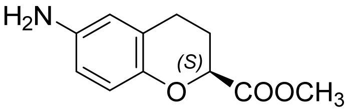	+39 (0.042, $CHCl_3$)	Not found	Wipf, P.; Huryn, D. M.; Laporte, M. G.; et al. **2022**, WO2022/120048.
24	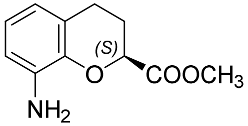	-18.1 (0.3, $CHCl_3$)	Not found	Wipf, P.; Huryn, D. M.; Laporte, M. G.; et al. **2022**, WO2022/120048.

Table S4. Conformational analysis of compound **35** at different levels in DMF.

	Carboxyl arrangement	Boltzmann distribution (%)														
		B3LYP			M062X		ωB97XD		APFD		BH&HLYP		O3LYP		Cam-B3LYP	
		6-311 G(d,p)	6-311+ G(d,p)	TZVP	6-311+ G(d,p)	TVZP	6-311+ G(d,p)	TZVP	6-311+ G(d,p)	TZVP	6-311+ G(d,p)	TZVP	6-311+ G(d,p)	TZVP	6-311+ G(d,p)	TZVP
29C1	e	40.56	57.00	59.47	32.17	37.75	49.77	53.44	41.06	45.79	61.56	56.43	49.17	50.44	61.78	63.49
29C2	e	17.15	16.84	16.33	9.75	9.01	9.35	8.42	4.48	4.34	13.06	19.3	22.93	22.7	14.01	13.49
29C3	e	8.15	9.93	8.46	3.83	3.65	6.01	5.20	5.02	4.79	10.80	7.48	13.92	12.85	6.17	5.58
29C4	a	17.47	7.56	7.53	33.06	28.47	15.51	14.19	16.29	14.64	7.35	9.84	7.31	7.25	8.48	8.29
29C5	a	7.26	5.87	5.69	9.31	10.54	12.23	12.59	16.2	15.85	3.86	3.63	3.68	3.8	5.78	5.78
29C6	a	9.34	2.70	2.42	11.76	10.47	7.02	6.03	16.82	14.44	3.18	3.26	2.83	2.81	3.73	3.32
29C7	e	0.02	0.06	0.06	0.02	0.02	0.03	0.03	0.01	0.02	0.11	0.03	0.13	0.12	0.02	0.02
29C8	a	0.05	0.04	0.04	0.10	0.09	0.08	0.1	0.12	0.13	0.08	0.03	0.03	0.03	0.03	0.03
	e	65.88	83.83	84.32	45.77	50.43	65.16	67.09	50.57	54.94	85.53	83.24	86.15	86.11	81.98	82.58
	a	34.12	16.17	15.68	54.23	49.57	34.84	32.91	49.43	45.06	14.47	16.76	13.85	13.89	18.02	17.42
Calc. $[\alpha]_D$[a]		-8.51	-1.13	-2.09	-31.86	-34.33	-26.09	-26.69	-44.89	-42.15	+3.08	+3.40	+4.83	+3.23	-4.03	-4.48
Calc. $[\alpha]_D$[b]		-6.08	+5.39	+4.76	-31.55	-33.30	-22.36	-22.31	-43.46	-39.36	+10.76	+10.97	+12.23	+10.99	+3.59	+3.58

[a] OR step at the B3LYP/Aug-cc-pVDZ level using PCM; [b] OR step at the M06-2X/Aug-cc-pVDZ level using PCM.

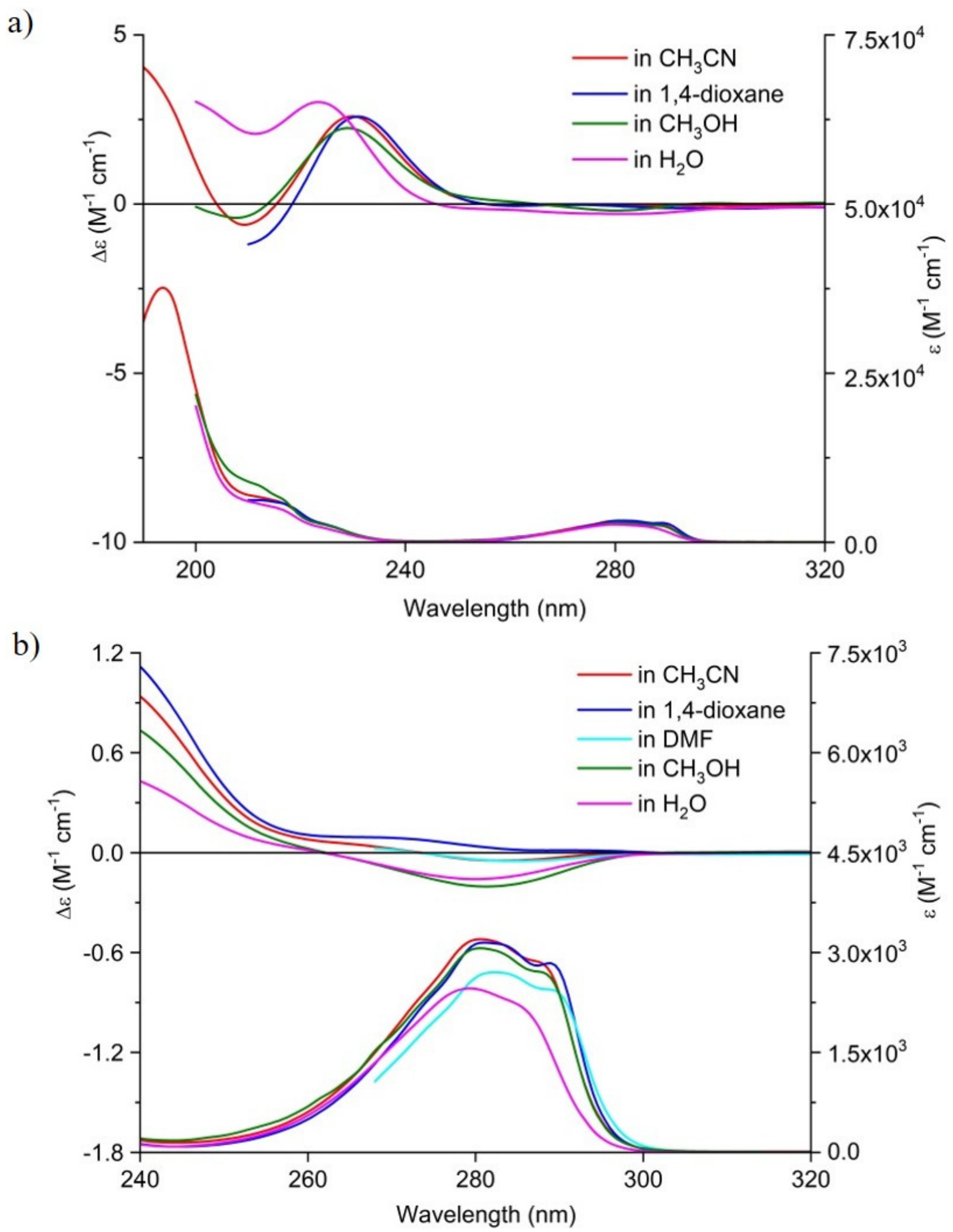

Figure S1. Experimental UV and ECD spectra of **35** in various solvents. a) full spectra from 320 nm to 190 nm, b) partially enlarged curves at 240-320 nm.
